# Supplementary material for: Registration and publication of emergency and elective randomised controlled trials in surgery: a cohort study from trial registries
Source: BMJ Open. 2018 Jul 7;8(7):e021700. doi: 10.1136/bmjopen-2018-021700 (PMC6042627; doi:10.1136/bmjopen-2018-021700)
Supplement: Supplementary file 1 [file bmjopen-2018-021700supp001.pdf]

Sponsorship	Emergency surgery; Number (% of total)	Elective surgery; Number (% of total)	All Number (% of total)
**Industry**	5 (12.8)	112 (29.9)	117 (28.6)
**Hospital**	18 (46.2)	120 (32)	138 (33.3)
**University**	10 (25.6)	92(24.5)	102 (24.6)
**Government**	5 (12.8)	21 (5.6)	26 (6.3)
**Research institute**	1 (2.6)	30 (8.0)	31 (7.5)
**TOTAL**	39 (100)	375 (100)	414 (100)

Supplementary Table 1 Comparison of the origin of sponsorship between emergency and elective surgical trials. Decimals do not add up exactly to total due to rounding.
