# Supplementary material for: Registration and publication of emergency and elective randomised controlled trials in surgery: a cohort study from trial registries
Source: BMJ Open. 2018 Jul 7;8(7):e021700. doi: 10.1136/bmjopen-2018-021700 (PMC6042627; doi:10.1136/bmjopen-2018-021700)
Supplement: Author's manuscript [file bmjopen-2018-021700.draft_revisions.pdf]

BMJ Open

BMJ Open is committed to open peer review. As part of this commitment we make the peer review history of every article we publish publicly available.

When an article is published we post the peer reviewers' comments and the authors' responses online. We also post the versions of the paper that were used during peer review. These are the versions that the peer review comments apply to.

The versions of the paper that follow are the versions that were submitted during the peer review process. They are not the versions of record or the final published versions. They should not be cited or distributed as the published version of this manuscript.

BMJ Open is an open access journal and the full, final, typeset and author-corrected version of record of the manuscript is available on our site with no access controls, subscription charges or pay-per-view fees (http://bmjopen.bmj.com).

If you have any questions on BMJ Open's open peer review process please email info.bmjopen@bmj.com

BMJ Open

Registration and Publication of Emergency and Elective Surgical Trials

Journal:	*BMJ Open*
Manuscript ID	bmjopen-2018-021700
Article Type:	Research
Date Submitted by the Author:	12-Jan-2018

Complete List of Authors:	Morley, Rachael Lucia; University of Bristol School of Social and Community Medicine, Centre for Surgical Research; North Bristol NHS Trust<br>Edmondson, Matthew; University of Bristol School of Social and Community Medicine, Centre for Surgical Research; Bristol Royal Infirmary<br>Rowlands, Ceri; University of Bristol School of Social and Community Medicine, Centre for Surgical Research<br>Blazeby, Jane; University of Bristol, Bristol Centre for Surgical Research, School of Social and Community Medicine; Bristol Royal Infirmary<br>Hinchliffe, Robert; University of Bristol School of Social and Community Medicine, Centre for Surgical Research; North Bristol NHS Trust

Keywords:	SURGERY, EMERGENCY, HEALTH SERVICES ADMINISTRATION & MANAGEMENT

SCHOLARONE™
Manuscripts

Registration and Publication of Emergency and Elective Surgical Trials

Morley RL[1,2], Edmondson MJ[1,3], Rowlands C[1], Blazeby JM[1,3], Hinchliffe RJ[1,2]

[1] *University of Bristol School of Clinical Science, Bristol Centre for Surgical Research Bristol, UK*

[2] *North Bristol NHS Trust, Bristol, UK*

[3] *Bristol Royal Infirmary, Bristol, UK*

Corresponding author: Rachael Lucia Morley
rm17210@bristol.ac.uk
G06D, Canynge Hall, 39 Whatley Road, Bristol BS8 2PN

Key words SURGERY, EMERGENCY, HEALTH SERVICE ADMINSTRATION & MANAGEMENT

Word count 3095

For peer review only

Abstract

Word count: 299

Objectives Emergency surgical practice constitutes 50% of the workload for surgeons, but the evidence base to underpin this activity is weak. The reasons behind a lack of high quality randomised control trials in emergency surgery are unclear. This study aims to establish the differences between the registration, completion and publication of emergency and elective surgical trials.

Design The clinicaltrials.gov and ISRCTN.com trials registry databases were searched for randomised control trials between 12th July 2010 and 12th July 2012 using the keyword "surgery.' Publications were systematically searched for in Pubmed, MEDLINE and EMBASE.

Participants Results with no surgical interventions were excluded. The remaining results were manually categorised into 'emergency' or 'elective' and 'surgical' or 'adjunct' by two reviewers.

Primary outcome measures Number of randomised control trials (RCTs) registered in emergency versus elective surgery.

Secondary outcome measures Number of RCTs published in emergency versus elective surgery; reasons why trials remain unpublished; funding, sponsorship and impact of published articles; number of adjunct trials registered in emergency and elective surgery.

Results Some 2700 randomised trials were registered. 1173 trials were on a surgical population and of these, 414 trials were studying surgery. Only 9.4% (39/414) of surgical trials were in emergency surgery. The proportion of trials successfully published were similar between emergency and elective surgery (18/39, 46.2% vs 195/375, 52.0%; p=0.800). Unpublished emergency surgical trials were equally likely to be terminated early (7/21, 33.3% vs 28/180, 15.6%; p=0.184) compared with elective trials, with low accrual accounting for a similar majority in both groups (3/7, 42.9% vs 13/28, 46.4%; p=1.000). Unpublished trials in both groups were equally likely to still be planning publication (11/21, 52.4% vs 127/180, 70.1%; p=0.564).

Conclusion Fewer randomised clinical trials are registered in emergency compared with elective surgery. Once trials are registered both groups are equally likely to be published.

Strengths and limitations

- Two trials registry databases interrogated.
- Systematic extraction of data from trials registries by two researchers.
- Publication search allowed 63 months from trial registration.

For peer review only

Funding

This work was undertaken with the support of the MRC ConDuCT-II (Collaboration and innovation for Difficult and Complex randomised controlled Trials In Invasive procedures) Hub for Trials Methodology Research (MR/K025643/1)

This study was supported by the NIHR Biomedical Research Centre at the University Hospitals Bristol NHS Foundation Trust and the University of Bristol. The views expressed are those of the authors and not necessarily those of the UK National Health Service, National Institute for Health Research, or Department of Health.

Competing interests

The authors declare no competing interests.

Author Contribution

All authors played a substantial role in the design of the study. Morley RL collected data, synthesised results and wrote the manuscript. Edmondson MJ collected data and critically revised the manuscript. Rowlands C resolved any conflict in data collection and critically revised the manuscript. Blazeby JM critically revised the manuscript. Hinchliffe RJ resolved conflict in data collection and critically revised the manuscript. All authors gave final approval of the version to be published and agree to be held accountable for all aspects of the work.

For peer review only

Introduction

It is estimated that up to 50% of the workload of surgical specialties is in emergency surgical care.(1,2) In low and middle income countries, this figure rises to 60% of the workload.(3) Current evidence shows patients undergoing emergency surgery are three times more likely to die than those undergoing elective surgery. For those that survive, they are twice as likely to suffer a complication.(4,5) The UK Emergency Laparotomy Network recently revealed a variation in mortality from 3.6 to 41.7%.(6) Clearly, there is benefit to be gained from more research into emergency surgery to achieve better and more consistent outcomes.(7)

Clinical trials are essential for improving patient outcomes. A previous study has found that 52% of surgical randomised control trials (RCTs) remain unpublished.(8) There is currently no knowledge about the number of trials registered before publication in emergency surgery. Anecdotally, emergency surgical trials are considered more difficult to organise than their elective counterparts. Barriers to recruitment such as consent, randomisation, ethical and logistical issues can be more challenging in an emergency setting. However, it is not known whether these barriers preclude emergency surgical trials from publication any more often than elective trials.

The primary objective of this study was to determine the number of RCTs registered in emergency versus elective surgery. The secondary objectives were to compare rates of publication in emergency versus elective surgery; reasons why trials were unpublished; variation by specialty; impact, funding and sponsorship of published trials; and the number of adjunct trials published in emergency vs elective surgery.

For peer review only

Methods

Criteria for inclusion and exclusion

Trials registered on two trials databases over a 2-year period between 12th July 2010 and 11th July 2012 that satisfied the following criteria were included; randomised control trials, at least one arm studying a surgical intervention or intervention within the perioperative period, adult populations, any gender from anywhere in the world. There was no limitation to the comparative group or type of outcome.

Trials with no surgery or with an intervention outside the perioperative period were excluded. Single arm and non-randomised trials were not included as they would unlikely have quality outcome data. Paediatric trials were also excluded as they are likely to have their own difficulties in trial design owing to the nature of involving children.

Definitions

Surgical interventions were defined as those involving physically changing body tissues and organs through manual operation such as cutting, suturing, abrading or the use of lasers.(9) Within this definition, this study included negative pressure wound therapy but excluded basic wound dressings (e.g self-adhesive dressing). Adjunct trials were defined as any intervention on a population of patients in the perioperative period that would not otherwise be defined as surgery e.g. anaesthesia, post-op rehabilitation, adjuvant chemotherapy.

Emergency operations were defined as unplanned admissions where it was not possible to discharge the patient home before their operation. This definition was only used when emergency or elective surgery was not specifically mentioned in the inclusion/exclusion criteria and it was not clear from the type of operation studied.

Search Strategy

Two online clinical trials databases were searched, 'ClinicalTrials.gov,' and the 'International Standard Randomised Controlled Trial Number (ISRCTN) registry.'(10,11) The registry searches were performed on the same day, 31st October 2016. Search strategies for each registry respectively were as follows:

1. Keyword "surgery" for non-paediatric, phase II-IV, interventional studies registered between 12th July 2010 and 12th July 2012.

2. Keyword "surgery" for trials registered between 12th July 2010 and 12th July 2012 (paediatric trials were manually excluded)

The 12th July was chosen as it produced the most reliable search results in ClinicalTrials.gov. For randomised control trials, the average time from registration to completion of data collection and then publication is 24 and 27 months respectively (51 months in total).(12) The 2010-2012 period was chosen to allow registered trials sufficient time to complete data collection and publication.

For peer review only

Two clinical trials registries were used to capture a range of trials from around the world. This aimed to avoid some selection bias if there was an unknown preference for trials to register with a specific registry.

Expanding the keyword search to “surgery OR operation” did not return any more results on ClinicalTrials.gov, and returned disproportionally more on ISRCTN (4,290 compared to 483 for “surgery” alone). On further scrutiny, searching for “surgery OR operation” on ISRCTN database appeared to remove all other search parameters and results included studies assigned from 2000-2016. It was therefore felt ‘surgery’ gave the most relevant search results.

The searches were categorised (surgery vs adjunct, elective vs emergency or excluded) by two reviewers (RLM, ME). A pilot was performed where 50 titles were searched by both. Disagreements were discussed and confirmed with a senior researcher (CR and RJH). The full search was then performed with each academic trainee categorising half the trials. If registry data was unclear about the nature of operation, the publications were checked for further information. If this failed to provide adequate information, the corresponding authors were emailed (see email search). Those that could not be categorised and those including both elective and emergency patients within the same study group were excluded from the analysis. RLM and ME both also extracted data about speciality, sponsorship funding (industry, hospital, university, government, research institute) and funding (industry, hospital, university, government, research institute, charity or investigator). Funding categories were only listed on ISRCTN and not clinicaltrials.gov.

Publication search strategy

The publication search was performed on 19th-20th October 2017, allowing a minimum 63 months from registration to publication. Trials can manually link their publication to the trials registry database and on occasion they are automatically indexed via PubMed. If this was not the case then a systematic search using Healthcare Databases Advanced Search (HDAS) was performed (searching PubMed, Medline and EMBASE). This was repeated in Google to identify any non-indexed publications (e.g. conference abstracts). Searches were performed using; trial registration number, study title, authors, institutions and key words. Papers were matched to studies according to trial design, interventions, recruitment numbers, dates of recruitment and hypotheses.(8)

Trials were considered as published if the data appeared in journals or in full or abstract form. Published protocols or experiences from the trials were not counted. If it was not found by the above search, data published on the clinical trials website was included as a publication.

Citation numbers were found by searching for publication title on the Web of Science Core Collection Database to indicate impact of articles. These were used as an indicator of impact of the publications.

Email search

Emails were identified from those provided on the clinical trials database. If there was no email provided, a google search for the trial investigator was performed to identify their

For peer review only

academic or hospital email. A standardised email was sent to seek clarification of inclusion of emergency/elective patients if this remained unclear. A second standardised email was sent to all trials that were unpublished to seek clarification of reasons why as a multiple-choice question.

Statistical analysis

R software (R Core Team 2013. **R**: A language and environment for statistical computing. Vienna, Austria. ISBN 3-900051-07-0, URL http://www.R-project.org)(13) was used and the chi squared test utilised for analysis and comparison of data.

Results

The initial searches identified 2700 results in total. Some 1473 trials were initially excluded (Figure 1). Of the remaining 1227 inclusions, 423 were surgical trials and 804 were adjunct trials. Considering only surgical trials, 9 were excluded as they could not be defined as emergency or elective (n=4) or specifically included both emergency and elective patients (n=5). This gave a final total of 414 surgical trials in the analysis, of which 39 (9.4%) were emergency and 375 (90.6%) were elective trials.

Of the 414 included surgical trials, 213 (51.4%) were published. The publication of emergency surgical trials was similar to elective surgical trials (18/39, 46.2% vs 195/375, 52.0%; p=0.800).

The majority of publications were cited within trial registrations (n=110). A substantial proportion were identified by searching HDAS and Google (n=90). Few publications were found via email responses (n=4) or data published only on trial registrations (n=9).

In searching for publications and clarifications of definitions, 148 emails were sent. Outcomes from emails sent were; no response (n=108), undeliverable (n=13) or replied (n=27). Many trials had no contact available (n=69).

Of the 201 unpublished trials, 21 (10.4%) were emergency and 180 (89.6%) were elective surgery. Reasons for non-publication of emergency and elective trials respectively were; completed but awaiting data analysis/publication process (7/21, 33.3%; 62/180, 34.4%), ongoing data collection (4/21, 19.0%; 65/180, 36.1%), terminated trial (7/21, 33.3%; 28/180, 15.6%) and unknown (3/21, 14.3%; 25/180, 13.9%;).

Unpublished trials in both groups were equally likely to still be planning publication (awaiting data analysis or ongoing data collection, 11/21, 52.4% vs 127/180, 70.1%; p=0.564). Emergency surgical trials were equally likely to be terminated early (7/21, 28/180; p=0.184) compared with elective trials, with low accrual being the most common reason in both groups (3/7, 42.9% vs 13/28, 44.8%; p=1.000).

Of 804 adjunct trials registered, 45 were excluded as they could not be defined (n=29) or they included both elective and emergency patients (n=16). This left 759 adjunct trials that were analysed. Elective trials represented 96% (n=729) whilst emergency trials only contributed 4% (n=30). This paper did not consider the non-publication of adjunct trials.

For peer review only

There was variation across specialties in the number of emergency and elective trials registered and published (Table 1).

For peer review only

Speciality		Trials registered; Number (% of trials in specialty)	Trials published; Number (% of trials registered)
General Surgery (All)	Elective	111 (84.7)	59 (53.2)
	Emergency	20 (15.3)	13 (65.0)
Orthopaedics	Elective	86 (86.9)	38 (44.2)
	Emergency	13 (13.1)	3 (23.1)
General (Lower GI)	Elective	43 (84.3)	27 (62.8)
	Emergency	8 (15.7)	6 (75.0)
General (Upper GI)	Elective	41 (89.1)	17 (41.5)
	Emergency	5 (10.9)	3 (60.0)
Ophthalmology	Elective	34 (100)	22 (64.7)
	Emergency	0 (0)	-
Cardiothoracic	Elective	33 (97.1)	18 (64.3)
	Emergency	1 (2.9)	0 (0)
Obstetrics & Gynaecology	Elective	29 (96.7)	13 (44.8)
	Emergency	1 (3.3)	0 (0)
Neurosurgery	Elective	24 (92.3)	8 (33.3)
	Emergency	2 (7.7)	2 (100)
Vascular	Elective	18 (100)	11 (61.1)
	Emergency	0 (0)	-
General (Other)	Elective	10 (58.2)	7 (70.0)
	Emergency	7 (41.8)	4 (57.1)
General (Breast)	Elective	14 (100)	6 (42.9)
	Emergency	0 (0)	-
Urology	Elective	14 (100)	7 (50.0)
	Emergency	0 (0)	-
Plastics	Elective	11 (84.6)	8 (72.7)
	Emergency	2 (15.4)	0 (0)

Table 1 Trials by speciality (excluding specialities with less than ten trials found) showing proportion of elective vs emergency trials and the percentage of these that were published. General surgery (all) includes general (lower GI), general (upper GI), general (breast) and general (other).

Most trials were sponsored by hospitals. Of published trials, there was no significant differences in sponsorship (table 2). Most surgical trials were funded by government bodies (table 3).

For peer review only - http://bmjopen.bmj.com/site/about/guidelines.xhtml

SPONSORSHIP	EMERGENCY SURGERY	ELECTIVE SURGERY	ALL
INDUSTRY	5	112	117
HOSPITAL	18	120	138
UNIVERSITY	9	92	101
GOVERNMENT	4	21	25
RESEARCH INSTITUTE	1	30	31
TOTAL	37	375	412

Table 2 Comparison of the origin of sponsorship between emergency and elective surgical trials

FUNDING	EMERGENCY SURGERY	ELECTIVE SURGERY	ALL
INDUSTRY	0	15	15
HOSPITAL	2	18	20
UNIVERSITY	0	13	13
GOVERNMENT	5	18	23
RESEARCH INSTITUTE	1	7	8
CHARITY	0	9	9
INVESTIGATOR	0	3	3
TOTAL	8	83	91

Table 3 Comparison of the origin of funding between emergency and elective surgical trials

Citation numbers were similar in emergency (mean 21.75, range 0-98) and elective (mean 21.42, range 0-306) surgical trials.

Discussion

This study found that there are significantly fewer randomised clinical trials registered in emergency surgery. However, emergency surgical trials that were registered were equally as likely to be published as elective surgical trials. These publications also had similar numbers

For peer review only

of citations. Recruitment difficulties leading to early trial termination were encountered at a similar rate between elective and emergency studies. Similarly, there was no clear difference between citation numbers in emergency and elective trials, which was used as a marker for research impact. Variation between specialties revealed a comparable pattern to the overall picture.

Registration and publication of all surgical trials has previously been studied.(8) Emergency and elective trials were not studied separately, but found that 48% of all surgical trials remained published at 38 months. The current paper allowed a further 25 months minimum for our publication search and the overall publication rate was similar (51.5%). This suggests a relative plateau in the rate of publication of the trials beyond 38 months.

The discontinuation of RCTs in critical care has also been studied, which included seven surgical trials.(14) They found the percentage of 'acute care' trials (critical care or care within 24 hours of presentation) was 7%, and a similar publication rate between 'acute' and 'non-acute' trials. However, slow recruitment caused twice as many acute trials to be discontinued than non-acute trials. The methodology was based on comparing published protocols and publications, as opposed to trial registration and publications.

This study has several limitations. Emergency was a binary definition reflecting the wording used within the trials registries. There are inherent difficulties when trying to categorise patients this way, as some operations are 'urgent' but not 'emergency' (e.g. bone fractures). It is likely that within the trials searched, the definition of emergency varied. It is possible that this paper gives an overestimation of true emergency surgical trials, where 'urgent' trials have been upgraded to 'emergency' in attempting to define them.

The timescale of follow up in this study may underestimate the true number of published trials. The average time to publication is 51 months and these results are a minimum of 63 months after the trials were first registered. The comparison to previous trials and plateau of publication at 38 versus 63 months is discussed above. Allowing a greater time interval may allow a different conclusion, but would also limit applicability to current practice.

Although this paper demonstrates a lack of registration of emergency surgical trials, further work is required to elicit the reasons. Because emergency trials are traditionally perceived as more difficult to achieve, both clinicians and sponsors may be more hesitant to invest their resources. This study shows that emergency surgical trials are equally as likely to be published as elective surgical trials and are a similar investment risk to elective randomised control trials. Indeed, many issues around consent and randomisation in emergency situations are being overcome.(15–19) With this knowledge, clinicians, researchers and sponsors may feel reassured and inclined to be involved with emergency surgical trials.

Emergency surgery is under-represented in the literature, despite the high volume of workload it creates and poor outcomes. This study shows that registered emergency and elective surgical trials are equally as likely to be published with comparable recruitment issues. A perceived lack of success of emergency surgical trials should no longer prevent investment into studies that would positively impact outcomes in emergency surgery.

For peer review only

For peer review only

References

1. Gale SC, Shafi S, Dombrovskiy VY, Arumugam D, Crystal JS. The public health burden of emergency general surgery in the United States. J Trauma Acute Care Surg [Internet]. 2014 Aug [cited 2017 Aug 3];77(2):202–8. Available from: http://www.ncbi.nlm.nih.gov/pubmed/25058242

2. RCSEng. Emergency Surgery - Standards for unscheduled surgical care [Internet]. 2011 [cited 2017 Oct 5]. Available from: https://www.google.co.uk/url?sa=t&rct=j&q=&esrc=s&source=web&cd=3&cad=rja&uact=8&ved=0ahUKEwiltJHc-JfXAhXBHxoKHcrzC7EQFggyMAI&url=https%3A%2F%2Fwww.rcseng.ac.uk%2F-%2Fmedia%2Ffiles%2Frcs%2Fabout-rcs%2Fregional%2Frcs_emergency_surgery_2011_web.pdf&usg=AOvV

3. McCord C, Ozgediz D, Beard JH, Debas HT. General Surgical Emergencies [Internet]. Essential Surgery: Disease Control Priorities, Third Edition (Volume 1). The International Bank for Reconstruction and Development / The World Bank; 2015 [cited 2017 Jul 3]. Available from: http://www.ncbi.nlm.nih.gov/pubmed/26741004

4. Mullen MG, Michaels AD, Mehaffey JH, Guidry CA, Turrentine FE, Hedrick TL, et al. Risk Associated With Complications and Mortality After Urgent Surgery vs Elective and Emergency Surgery. JAMA Surg [Internet]. 2017 May 10 [cited 2017 Jul 3]; Available from: http://www.ncbi.nlm.nih.gov/pubmed/28492821

5. Visser A, Geboers B, Gouma DJ, Goslings JC, Ubbink DT. Predictors of surgical complications: A?systematic review. Surgery [Internet]. 2015 Jul [cited 2017 Jul 5];158(1):58–65. Available from: http://linkinghub.elsevier.com/retrieve/pii/S0039606015000288

6. Saunders DI, Murray D, Pichel AC, Varley S, Peden CJ, UK Emergency Laparotomy Network. Variations in mortality after emergency laparotomy: the first report of the UK Emergency Laparotomy Network. Br J Anaesth [Internet]. 2012 Sep 1 [cited 2017 Nov 13];109(3):368–75. Available from: http://www.ncbi.nlm.nih.gov/pubmed/22728205

7. Becher RD, Hoth JJ, Miller PR, Mowery NT, Chang MC, Meredith JW. A critical assessment of outcomes in emergency versus nonemergency general surgery using the American College of Surgeons National Surgical Quality Improvement Program database. Am Surg [Internet]. 2011 Jul [cited 2017 Oct 5];77(7):951–9. Available from: http://www.ncbi.nlm.nih.gov/pubmed/21944366

8. Chapman SJ, Shelton B, Mahmood H, Fitzgerald JE, Harrison EM, Bhangu A. Discontinuation and non-publication of surgical randomised controlled trials: observational study. BMJ [Internet]. 2014 Dec 9 [cited 2016 Oct 31];349(dec09_1):g6870. Available from: http://www.bmj.com/content/349/bmj.g6870

For peer review only

9. Cook JA. The challenges faced in the design, conduct and analysis of surgical randomised controlled trials. Trials [Internet]. 2009 Dec 6 [cited 2017 Oct 19];10(1):9. Available from: http://www.ncbi.nlm.nih.gov/pubmed/19200379

10. BioMed Central Ltd. ISRCTN Registry [Internet]. [cited 2016 Oct 31]. Available from: http://www.isrctn.com/

11. U.S. National Library of Medicine. ClinicalTrials.gov [Internet]. [cited 2016 Oct 31]. Available from: https://clinicaltrials.gov/

12. Walker KF, Stevenson G, Thornton JG. Discrepancies between registration and publication of randomised controlled trials: an observational study. JRSM open [Internet]. SAGE Publications; 2014 May 1 [cited 2016 Oct 31];5(5):2042533313517688. Available from: http://shr.sagepub.com/content/5/5/2042533313517688.full

13. R Development Core Team (2008). A language and environment for statistical computing. R Foundation for Statistical Computing, Vienna, Austria [Internet]. p. http://www.r-project.org [accessed 13th Nov 2017]. Available from: http://www.r-project.org

14. Schandelmaier S, von Elm E, You JJ, Blümle A, Tomonaga Y, Lamontagne F, et al. Premature Discontinuation of Randomized Trials in Critical and Emergency Care. Crit Care Med [Internet]. 2016 Jan [cited 2017 Nov 13];44(1):130–7. Available from: http://www.ncbi.nlm.nih.gov/pubmed/26468895

15. Edwards M, Grocott M, Pearse R. Study Documents - FLuid Optimisation in Emergency LAparotomy trial [Internet]. 2017 [cited 2017 Nov 13]. Available from: http://www.floela.org/documents

16. Jansen J, Campbell M. UK-REBOA [Internet]. 2017 [cited 2017 Nov 13]. Available from: https://w3.abdn.ac.uk/hsru/REBOA/Public/Public/index.cshtml

17. Taylor J, Black S, J. Brett S, Kirby K, Nolan JP, Reeves BC, et al. Design and implementation of the AIRWAYS-2 trial: A multi-centre cluster randomised controlled trial of the clinical and cost effectiveness of the i-gel supraglottic airway device versus tracheal intubation in the initial airway management of out of hospital cardiac arrest. Resuscitation [Internet]. 2016 Dec [cited 2017 Nov 13];109:25–32. Available from: http://www.ncbi.nlm.nih.gov/pubmed/27697605

18. Roberts I, Shakur H, Coats T, Hunt B, Balogun E, Barnetson L, et al. The CRASH-2 trial: a randomised controlled trial and economic evaluation of the effects of tranexamic acid on death, vascular occlusive events and transfusion requirement in bleeding trauma patients. Heal Technol Assess [Internet]. 2013 Mar [cited 2017 Aug 3];17(10):1–79. Available from: http://www.ncbi.nlm.nih.gov/pubmed/23477634

19. IMPROVE Trial Investigators I trial, Powell JT, Sweeting MJ, Thompson MM, Ashleigh R, Bell R, et al. Endovascular or open repair strategy for ruptured abdominal aortic

For peer review only

aneurysm: 30 day outcomes from IMPROVE randomised trial. BMJ [Internet]. British Medical Journal Publishing Group; 2014 Jan 13 [cited 2017 Nov 13];348:f7661. Available from: http://www.ncbi.nlm.nih.gov/pubmed/24418950

For peer review only

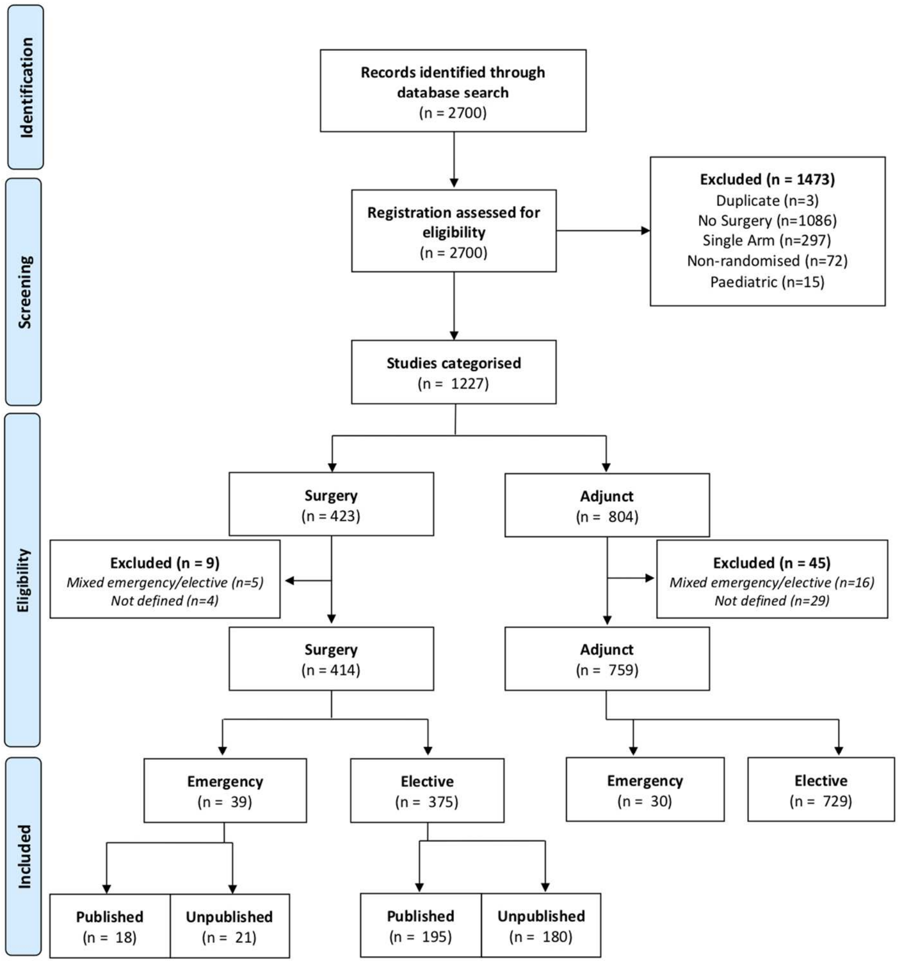

Figure 1 PRISMA style flow diagram of search results, exclusions and group numbers.

215x279mm (150 x 150 DPI)

BMJ Open

Registration and Publication of Emergency and Elective Surgical Trials: A Systematic Review of Trials Registries

Journal:	*BMJ Open*
Manuscript ID	bmjopen-2018-021700.R1
Article Type:	Research
Date Submitted by the Author:	03-Apr-2018

Complete List of Authors:	Morley, Rachael Lucia; University of Bristol School of Social and Community Medicine, Centre for Surgical Research; North Bristol NHS Trust<br>Edmondson, Matthew; University of Bristol School of Social and Community Medicine, Centre for Surgical Research; Bristol Royal Infirmary<br>Rowlands, Ceri; University of Bristol School of Social and Community Medicine, Centre for Surgical Research<br>Blazeby, Jane; University of Bristol, Bristol Centre for Surgical Research, School of Social and Community Medicine; Bristol Royal Infirmary<br>Hinchliffe, Robert; University of Bristol School of Social and Community Medicine, Centre for Surgical Research; North Bristol NHS Trust

**Primary Subject Heading**:	Surgery
Secondary Subject Heading:	Emergency medicine, Health policy
Keywords:	SURGERY, EMERGENCY, HEALTH SERVICES ADMINISTRATION & MANAGEMENT

SCHOLARONE™ Manuscripts

Registration and Publication of Emergency and Elective Surgical Trials: A Systematic Review of Trials Registries

Morley RL[1,2], Edmondson MJ[1,3], Rowlands C[1], Blazeby JM[1,3], Hinchliffe RJ[1,2]

[1] Bristol Centre for Surgical Research, Bristol Medical School, University of Bristol, Bristol, UK

[2] North Bristol NHS Trust, Bristol, UK

[3] Bristol Royal Infirmary, Bristol, UK

Corresponding author: Rachael Lucia Morley
rm17210@bristol.ac.uk
G06D, Canynge Hall, 39 Whatley Road, Bristol BS8 2PN

Key words SURGERY, EMERGENCY, HEALTH SERVICE ADMINSTRATION & MANAGEMENT

Word count 2169

For peer review only

Abstract

Word count: 297

Objectives Emergency surgical practice constitutes 50% of the workload for surgeons, but there is a lack of high quality randomised control trials in emergency surgery. This study aims to establish the differences between the registration, completion and publication of emergency and elective surgical trials.

Design The clinicaltrials.gov and ISRCTN.com trials registry databases were searched for randomised control trials between 12th July 2010 and 12th July 2012 using the keyword "surgery.' Publications were systematically searched for in Pubmed, MEDLINE and EMBASE.

Participants Results with no surgical interventions were excluded. The remaining results were manually categorised into 'emergency' or 'elective' and 'surgical' or 'adjunct' by two reviewers.

Primary outcome measures Number of randomised control trials (RCTs) registered in emergency versus elective surgery.

Secondary outcome measures Number of RCTs published in emergency versus elective surgery; reasons why trials remain unpublished; funding, sponsorship and impact of published articles; number of adjunct trials registered in emergency and elective surgery.

Results 2700 randomised trials were registered. 1173 trials were on a surgical population and of these, 414 trials were studying surgery. Only 9.4% (39/414) of surgical trials were in emergency surgery. The proportion of trials successfully published were similar between emergency and elective surgery (46.2% vs 52.0%; Odds ratio 0.51, 95%CI 0.24 - 1.09). Unpublished emergency surgical trials were equally likely to be terminated early compared with elective trials (33.3% vs 15.6%; OR 2.4, 95%CI 0.88 - 6.40). Low accrual accounted for a similar majority in both groups (42.9% vs 44.8%; OR 0.89, 95%CI 0.17 - 4.74). Unpublished trials in both groups were equally likely to still be planning publication (52.4% vs 70.1%; OR 0.50, 95%CI 0.20 - 1.25).

Conclusion Fewer randomised clinical trials are registered in emergency than elective surgery. Once trials are registered both groups are equally likely to be published.

Article Summary

Strengths and limitations

- Two trials registry databases interrogated giving a large number of registrations.
- Systematic extraction of data from trials registries by two researchers.
- Publication search allowed 63 months from trial registration.

For peer review only

Funding

This work was undertaken with the support of the MRC ConDuCT-II (Collaboration and innovation for Difficult and Complex randomised controlled Trials In Invasive procedures) Hub for Trials Methodology Research (MR/K025643/1)

This study was supported by the NIHR Biomedical Research Centre at the University Hospitals Bristol NHS Foundation Trust and the University of Bristol. The views expressed are those of the authors and not necessarily those of the UK National Health Service, National Institute for Health Research, or Department of Health.

Competing interests

The authors declare no competing interests.

Author Contribution

All authors played a substantial role in the design of the study. Morley RL collected data, synthesised results and wrote the manuscript. Edmondson MJ collected data and critically revised the manuscript. Rowlands C resolved any conflict in data collection and critically revised the manuscript. Blazeby JM critically revised the manuscript. Hinchliffe RJ resolved conflict in data collection and critically revised the manuscript. All authors gave final approval of the version to be published and agree to be held accountable for all aspects of the work.

Data Sharing Statement

Data is available upon request from the corresponding author.

For peer review only

Introduction

It is estimated that up to 50% of the workload of surgical specialties is in emergency surgical care.(1,2) In low and middle income countries, this figure rises to 60% of the workload.(3) Current evidence shows patients undergoing emergency surgery are three times more likely to die than those undergoing elective surgery. For those that survive, they are twice as likely to suffer a complication.(4,5) The UK Emergency Laparotomy Network recently revealed a variation in mortality from 3.6 to 41.7%.(6) Clearly, there is benefit to be gained from more research into emergency surgery to achieve better and more consistent outcomes.(7)

Clinical trials are essential for improving patient outcomes. A previous study has found that 52% of surgical randomised control trials (RCTs) remain unpublished.(8) There is currently no knowledge about the number of trials registered before publication in emergency surgery. Anecdotally, emergency surgical trials are considered more difficult to organise than their elective counterparts. Barriers to recruitment such as consent, randomisation, ethical and logistical issues can be more challenging in an emergency setting. However, it is not known whether these barriers preclude emergency surgical trials from publication any more often than elective trials.

The primary objective of this study was to determine the number of RCTs registered in emergency versus elective surgery. The secondary objectives were to compare rates of publication in emergency versus elective surgery; reasons why trials were unpublished; variation by specialty; impact, funding and sponsorship of published trials; and the number of adjunct trials published in emergency vs elective surgery.

For peer review only

Methods

Criteria for inclusion and exclusion

Trials registered on two trials databases over a 2-year period between 12th July 2010 and 11th July 2012 that satisfied the following criteria were included; randomised control trials, at least one arm studying a surgical intervention or intervention within the perioperative period, adult populations, any gender from anywhere in the world. There was no limitation to the comparative group or type of outcome.

Trials with no surgery or with an intervention outside the perioperative period were excluded. Single arm and non-randomised trials were not included as they would unlikely have quality outcome data. Paediatric trials were also excluded as they are likely to have their own difficulties in trial design owing to the nature of involving children.

Definitions

Surgical interventions were defined as those involving physically changing body tissues and organs through manual operation such as cutting, suturing, abrading or the use of lasers.(9) Within this definition, this study included negative pressure wound therapy but excluded basic wound dressings (e.g self-adhesive dressing). Adjunct trials were defined as any intervention on a population of patients in the perioperative period that would not otherwise be defined as surgery e.g. anaesthesia, post-op rehabilitation, adjuvant chemotherapy.

Emergency operations were defined as unplanned admissions where it was not possible to discharge the patient home before their operation. This definition was only used when emergency or elective surgery was not specifically mentioned in the inclusion/exclusion criteria and it was not clear from the type of operation studied.

Search Strategy

Two online clinical trials databases were searched, 'ClinicalTrials.gov,' and the 'International Standard Randomised Controlled Trial Number (ISRCTN) registry.'(10,11) The registry searches were performed on the same day, 31st October 2016. Search strategies for each registry respectively were as follows:

1. Keyword "surgery" for non-paediatric, phase II-IV, interventional studies registered between 12th July 2010 and 12th July 2012.

2. Keyword "surgery" for trials registered between 12th July 2010 and 12th July 2012 (paediatric trials were manually excluded)

For randomised control trials, the average time from registration to completion of data collection and then publication is 24 and 27 months respectively (51 months in total).(12) The 2010-2012 period was chosen to allow registered trials sufficient time to complete data collection and publication.

Two clinical trials registries were used to capture a range of trials from around the world. This aimed to avoid some selection bias if there was an unknown preference for trials to register with a specific registry.

For peer review only

Expanding the keyword search to “surgery OR operation” did not return any more results on ClinicalTrials.gov and returned disproportionally more on ISRCTN (4,290 compared to 483 for “surgery” alone). On further scrutiny, searching for “surgery OR operation” on ISRCTN database appeared to remove all other search parameters and results included studies assigned from 2000-2016. It was therefore felt ‘surgery’ gave the most relevant search results.

The searches were categorised (surgery vs adjunct, elective vs emergency or excluded) by two reviewers (RLM, ME). A pilot was performed where 50 titles were searched by both. Disagreements were discussed and confirmed with a senior researcher (CR and RJH). The full search was then performed with each academic trainee categorising half the trials. If registry data was unclear about the nature of operation, the publications were checked for further information. If this failed to provide adequate information, the corresponding authors were emailed (see email search). Those that could not be categorised and those including both elective and emergency patients within the same study group were excluded from the analysis. RLM and ME both also extracted data about speciality, sponsorship (industry, hospital, university, government, research institute) and funding (industry, hospital, university, government, research institute, charity or investigator).

Publication search strategy

The publication search was performed on 19th-20th October 2017, allowing a minimum 63 months from registration to publication. Trials can manually link their publication to the trials registry database and on occasion they are automatically indexed via PubMed. If this was not the case then a systematic search using Healthcare Databases Advanced Search (HDAS) was performed (selecting PubMed, Medline and EMBASE). This was repeated in Google to identify any non-indexed publications (e.g. conference abstracts). Searches were performed using; trial registration number, study title, authors, institutions and key words. Papers were matched to studies according to trial design, interventions, recruitment numbers, dates of recruitment and hypotheses.(8)

Trials were considered as published if the data appeared in journals or in full or abstract form. Published protocols or experiences from the trials were not counted. Some trials publish their data directly to the trials website and this was counted as a publication if no other publication was found.

Citation numbers were found by searching for publication title on the Web of Science Core Collection Database to indicate impact of articles. These were used as an indicator of impact of the publications.

Email search

Emails were identified from those provided on the clinical trials database. If there was no email provided, a google search for the trial investigator was performed to identify their academic or hospital email. Two standardised emails were sent. The first was sent to seek clarification of inclusion of emergency/elective patients if this remained unclear. The second email was sent to all unpublished trials. This clarified publication status and if unpublished, a multiple-choice question of reasons why.

For peer review only

Statistical analysis

R software (R Core Team 2013. **R**: A language and environment for statistical computing. Vienna, Austria. ISBN 3-900051-07-0, URL http://www.R-project.org)(13) was used. A paired t-test was used to compare citation numbers and odds ratios used all other comparisons at 95% confidence intervals.

Patient and Public Involvement

No patients were involved in the design or implementation of this study.

Results

The initial searches identified 2700 results in total. 1473 trials were initially excluded (Figure 1). Of the remaining 1227 inclusions, 423 were surgical trials and 804 were adjunct trials. Considering only surgical trials, 9 were excluded as they could not be defined as emergency or elective (n=4) or specifically included both emergency and elective patients (n=5). This gave a final total of 414 surgical trials in the analysis, of which 39 (9.4%) were emergency and 375 (90.6%) were elective trials.

Of the 414 included surgical trials, 213 (51.4%) were published. The publication of emergency surgical trials was similar to elective surgical trials (46.2% vs 52.0%; Odds ratio 0.51, 95%CI 0.24 - 1.09). The rate of publications, in each group and overall, are shown in figure 2. There was variation across specialties in the number of emergency and elective surgical trials registered and published (Table 1). Citation numbers were similar in emergency (mean 21.75, range 0-98) and elective (mean 21.59, range 0-306) surgical trials (difference 0.16 95%CI -16.7 to 16.9).

Speciality		Trials registered; Number (% of trials in specialty)	Trials published; Number (% of trials registered)
**General Surgery (All)**	Elective	111 (84.7)	59 (53.2)
	Emergency	20 (15.3)	13 (65.0)
**Orthopaedics**	Elective	86 (86.9)	38 (44.2)
	Emergency	13 (13.1)	3 (23.1)
**General (Lower GI)***	Elective	43 (84.3)	27 (62.8)
	Emergency	8 (15.7)	6 (75.0)
**General (Upper GI)***	Elective	41 (89.1)	17 (41.5)
	Emergency	5 (10.9)	3 (60.0)
**Ophthalmology**	Elective	34 (100)	22 (64.7)
	Emergency	0 (0)	-
**Cardiothoracic**	Elective	33 (97.1)	18 (64.3)
	Emergency	1 (2.9)	0 (0)
**Obstetrics & Gynaecology**	Elective	29 (96.7)	13 (44.8)
	Emergency	1 (3.3)	0 (0)
**Neurosurgery**	Elective	24 (92.3)	8 (33.3)
	Emergency	2 (7.7)	2 (100)

For peer review only

**Vascular**	Elective	18 (100)	11 (61.1)
	Emergency	0 (0)	-
**General (Other)***	Elective	10 (58.2)	7 (70.0)
	Emergency	7 (41.8)	4 (57.1)
**General (Breast)***	Elective	14 (100)	6 (42.9)
	Emergency	0 (0)	-
**Urology**	Elective	14 (100)	7 (50.0)
	Emergency	0 (0)	-
**Plastics**	Elective	11 (84.6)	8 (72.7)
	Emergency	2 (15.4)	0 (0)
**TOTAL**	Elective	360 (100)	184 (51.1)
	Emergency	39 (100)	18 (46.2)

*Table 1 Surgical trials by speciality (excluding specialities with less than ten trials found) showing proportion of elective vs emergency trials and the percentage of these that were published. * Categories combine into general surgery (all). Only general surgery (all) was included in the totals*

The majority of these publications were cited within trial registrations (n=110). A substantial proportion were identified by searching HDAS and Google (n=90). Few publications were found via email responses (n=4) or data published only on trial registrations (n=9).

In searching for publications and clarifications of definitions, 148 emails were sent. Outcomes from emails sent were; no response (n=108), undeliverable (n=13) or replied (n=27). Many trials had no contact available (n=69).

Of the 201 unpublished trials, 21 (10.4%) were emergency and 180 (89.6%) were elective surgery. Reasons for non-publication of emergency and elective trials respectively were; completed but awaiting data analysis/publication process (7/21, 33.3%; 62/180, 34.4%), ongoing data collection (4/21, 19.0%; 65/180, 36.1%), terminated trial (7/21, 33.3%; 28/180, 15.6%) and unknown (3/21, 14.3%; 25/180, 13.9%;).

Unpublished trials in both groups were equally likely to still be planning publication (awaiting data analysis or ongoing data collection; 52.4% vs 70.1%; OR 0.50, 95%CI 0.20 - 1.25). Unpublished emergency surgical trials were equally likely to be terminated early compared with elective trials (33.3% vs 15.6%; OR 2.4, 95%CI 0.88 - 6.40). Low accrual accounted for a similar majority in both groups (42.9% vs 44.8%; OR 0.89, 95%CI 0.17 - 4.74).

For peer review only

	Emergency surgery; Number (% of total)	Elective surgery; Number (% of total)	All Number (%)
**Completed and awaiting publication**	7 (33.3)	62 (34.4)	69 (34.3)
**Ongoing data collection**	4 (19.0)	65 (36.1)	69 (34.3)
**Terminated**	7 (33.3)	29 (15.6)	36 (17.9)
**Unknown**	3 (14.3)	24 (13.9)	27 (13.4)
**TOTAL**	21 (100)	180 (100)	201 (100)

Table 2 Reasons for trials remaining unpublished. Decimals do not add up exactly to total due to rounding.

Funding categories were only listed on ISRCTN and not clinicaltrials.gov. Not all trials registered listed sponsorship. Most trials were sponsored by hospitals. Of published trials, there was no significant differences in sponsorship (supplementary table 1). Most surgical trials were funded by government bodies (table 3).

Funding	Emergency surgery; Number (% of total)	Elective surgery; Number (% of total)	All Number (%)
**Industry**	0 (0)	15 (18.1)	15 (16.5)
**Hospital**	2 (25.0)	18 (21.7)	20 (22.0)
**University**	0 (0)	13 (15.7)	13 (14.3)
**Government**	5 (62.5)	18 (21.7)	23 (25.3)
**Research institute**	1 (12.5)	7 (8.4)	8 (8.8)
**Charity**	0 (0)	9 (10.8)	9 (10.0)
**Investigator**	0 (0)	3 (3.6)	3 (3.3)
**Total**	8 (100)	83 (100)	91 (100)

Table 3 Comparison of the origin of funding between emergency and elective surgical trials. This data was only available from ISRCTN and not clinicaltrials.gov. Decimals do not add up exactly to total due to rounding.

Of 804 adjunct trials registered, 45 were excluded as they could not be defined (n=29) or they included both elective and emergency patients (n=16). This left 759 adjunct trials that

For peer review only

were analysed. Elective trials represented 96% (n=729) whilst emergency trials only contributed 4% (n=30). This paper did not consider the non-publication of adjunct trials.

Discussion

This study found that there are significantly fewer randomised clinical trials registered in emergency surgery. However, emergency surgical trials that were registered were equally as likely to be published as elective surgical trials. Recruitment difficulties leading to early trial termination were encountered at a similar rate between elective and emergency studies. Similarly, there was no clear difference between citation numbers in emergency and elective trials, which was used as a marker for research impact. Variation between specialties revealed a comparable pattern to the overall picture.

Registration and publication of all surgical trials has previously been studied.(8) Emergency and elective trials were not studied separately, but found that 48% of all surgical trials remained published at 38 months. The current paper allowed a further 25 months minimum for our publication search and the overall publication rate was similar (51.5%). This suggests a relative plateau, in the rate of publication of the trials beyond 38 months. Interpretation of figure 2 however sees a plateau at around 54 months. Whilst more publications could be expected after this time, extending the search interval may limit applicability to current practice.

The discontinuation of RCTs in critical care has also been studied, which included seven surgical trials.(14) They found the percentage of ‘acute care’ trials (critical care or care within 24 hours of presentation) was 7%, and a similar publication rate between ‘acute’ and ‘non-acute’ trials. However, slow recruitment caused twice as many acute trials to be discontinued than non-acute trials. The methodology was based on comparing published protocols and publications, as opposed to trial registration and publications.

This study has several limitations. Emergency was a binary definition reflecting the wording used within the trials registries. There are inherent difficulties when trying to categorise patients this way, as some operations are 'urgent' but not 'emergency' (e.g. bone fractures). It is likely that within the trials searched, the definition of emergency varied. It is possible that this paper gives an overestimation of true emergency surgical trials, where 'urgent' trials have been upgraded to 'emergency' in attempting to define them.

Although this paper demonstrates a lack of registration of emergency surgical trials compared to surgical workload, further work is required to elicit the reasons. Because emergency trials are traditionally perceived as more difficult to achieve, both clinicians and sponsors may be more hesitant to invest their resources. This study shows that emergency surgical trials are equally as likely to be published as elective surgical trials and are a similar investment risk to elective randomised control trials. Indeed, many issues around consent and randomisation in emergency situations are being overcome.(15–19) With this knowledge, clinicians, researchers and sponsors may feel reassured and inclined to be involved with emergency surgical trials.

For peer review only

Emergency surgery is under-represented in the literature, despite the high volume of workload it creates and poor outcomes. This study shows that registered emergency and elective surgical trials are equally as likely to be published with comparable recruitment issues. A perceived lack of success of emergency surgical trials should no longer prevent investment into studies that would positively impact outcomes in emergency surgery.

For peer review only

Figure Legend

Figure 1. PRISMA style flow diagram of search results, exclusions and group numbers.

Figure 2. Kaplan-Meier curves showing publication rates of: A emergency surgical trials B elective surgical trials and C all surgical trials. Only trials published on or after the July 2012 were included, there is a cohort of trials that are registered after they started/published.

For peer review only

References

1. Gale SC, Shafi S, Dombrovskiy VY, Arumugam D, Crystal JS. The public health burden of emergency general surgery in the United States. J Trauma Acute Care Surg [Internet]. 2014 Aug [cited 2017 Aug 3];77(2):202–8. Available from: http://www.ncbi.nlm.nih.gov/pubmed/25058242

2. RCSEng. Emergency Surgery - Standards for unscheduled surgical care [Internet]. 2011 [cited 2017 Oct 5]. Available from: https://www.google.co.uk/url?sa=t&rct=j&q=&esrc=s&source=web&cd=3&cad=rja&uact=8&ved=0ahUKEwiltJHc-JfXAhXBHxoKHcrzC7EQFggyMAI&url=https%3A%2F%2Fwww.rcseng.ac.uk%2F-%2Fmedia%2Ffiles%2Frcs%2Fabout-rcs%2Fregional%2Frcs_emergency_surgery_2011_web.pdf&usg=AOvV

3. McCord C, Ozgediz D, Beard JH, Debas HT. General Surgical Emergencies [Internet]. Essential Surgery: Disease Control Priorities, Third Edition (Volume 1). The International Bank for Reconstruction and Development / The World Bank; 2015 [cited 2017 Jul 3]. Available from: http://www.ncbi.nlm.nih.gov/pubmed/26741004

4. Mullen MG, Michaels AD, Mehaffey JH, Guidry CA, Turrentine FE, Hedrick TL, et al. Risk Associated With Complications and Mortality After Urgent Surgery vs Elective and Emergency Surgery. JAMA Surg [Internet]. 2017 May 10 [cited 2017 Jul 3]; Available from: http://www.ncbi.nlm.nih.gov/pubmed/28492821

5. Visser A, Geboers B, Gouma DJ, Goslings JC, Ubbink DT. Predictors of surgical complications: A?systematic review. Surgery [Internet]. 2015 Jul [cited 2017 Jul 5];158(1):58–65. Available from: http://linkinghub.elsevier.com/retrieve/pii/S0039606015000288

6. Saunders DI, Murray D, Pichel AC, Varley S, Peden CJ, UK Emergency Laparotomy Network. Variations in mortality after emergency laparotomy: the first report of the UK Emergency Laparotomy Network. Br J Anaesth [Internet]. 2012 Sep 1 [cited 2017 Nov 13];109(3):368–75. Available from: http://www.ncbi.nlm.nih.gov/pubmed/22728205

7. Becher RD, Hoth JJ, Miller PR, Mowery NT, Chang MC, Meredith JW. A critical assessment of outcomes in emergency versus nonemergency general surgery using the American College of Surgeons National Surgical Quality Improvement Program database. Am Surg [Internet]. 2011 Jul [cited 2017 Oct 5];77(7):951–9. Available from: http://www.ncbi.nlm.nih.gov/pubmed/21944366

8. Chapman SJ, Shelton B, Mahmood H, Fitzgerald JE, Harrison EM, Bhangu A. Discontinuation and non-publication of surgical randomised controlled trials: observational study. BMJ [Internet]. 2014 Dec 9 [cited 2016 Oct 31];349(dec09_1):g6870. Available from: http://www.bmj.com/content/349/bmj.g6870

For peer review only

9. Cook JA. The challenges faced in the design, conduct and analysis of surgical randomised controlled trials. Trials [Internet]. 2009 Dec 6 [cited 2017 Oct 19];10(1):9. Available from: http://www.ncbi.nlm.nih.gov/pubmed/19200379

10. BioMed Central Ltd. ISRCTN Registry [Internet]. [cited 2016 Oct 31]. Available from: http://www.isrctn.com/

11. U.S. National Library of Medicine. ClinicalTrials.gov [Internet]. [cited 2016 Oct 31]. Available from: https://clinicaltrials.gov/

12. Walker KF, Stevenson G, Thornton JG. Discrepancies between registration and publication of randomised controlled trials: an observational study. JRSM open [Internet]. SAGE Publications; 2014 May 1 [cited 2016 Oct 31];5(5):2042533313517688. Available from: http://shr.sagepub.com/content/5/5/2042533313517688.full

13. R Development Core Team (2008). A language and environment for statistical computing. R Foundation for Statistical Computing, Vienna, Austria [Internet]. p. http://www.r-project.org [accessed 13th Nov 2017]. Available from: http://www.r-project.org

14. Schandelmaier S, von Elm E, You JJ, Blümle A, Tomonaga Y, Lamontagne F, et al. Premature Discontinuation of Randomized Trials in Critical and Emergency Care. Crit Care Med [Internet]. 2016 Jan [cited 2017 Nov 13];44(1):130–7. Available from: http://www.ncbi.nlm.nih.gov/pubmed/26468895

15. Edwards M, Grocott M, Pearse R. Study Documents - FLuid Optimisation in Emergency LAparotomy trial [Internet]. 2017 [cited 2017 Nov 13]. Available from: http://www.floela.org/documents

16. Jansen J, Campbell M. UK-REBOA [Internet]. 2017 [cited 2017 Nov 13]. Available from: https://w3.abdn.ac.uk/hsru/REBOA/Public/Public/index.cshtml

17. Taylor J, Black S, J. Brett S, Kirby K, Nolan JP, Reeves BC, et al. Design and implementation of the AIRWAYS-2 trial: A multi-centre cluster randomised controlled trial of the clinical and cost effectiveness of the i-gel supraglottic airway device versus tracheal intubation in the initial airway management of out of hospital cardiac arrest. Resuscitation [Internet]. 2016 Dec [cited 2017 Nov 13];109:25–32. Available from: http://www.ncbi.nlm.nih.gov/pubmed/27697605

18. Roberts I, Shakur H, Coats T, Hunt B, Balogun E, Barnetson L, et al. The CRASH-2 trial: a randomised controlled trial and economic evaluation of the effects of tranexamic acid on death, vascular occlusive events and transfusion requirement in bleeding trauma patients. Heal Technol Assess [Internet]. 2013 Mar [cited 2017 Aug 3];17(10):1–79. Available from: http://www.ncbi.nlm.nih.gov/pubmed/23477634

19. IMPROVE Trial Investigators I trial, Powell JT, Sweeting MJ, Thompson MM, Ashleigh R, Bell R, et al. Endovascular or open repair strategy for ruptured abdominal aortic

For peer review only

aneurysm: 30 day outcomes from IMPROVE randomised trial. BMJ [Internet]. British Medical Journal Publishing Group; 2014 Jan 13 [cited 2017 Nov 13];348:f7661. Available from: http://www.ncbi.nlm.nih.gov/pubmed/24418950

For peer review only

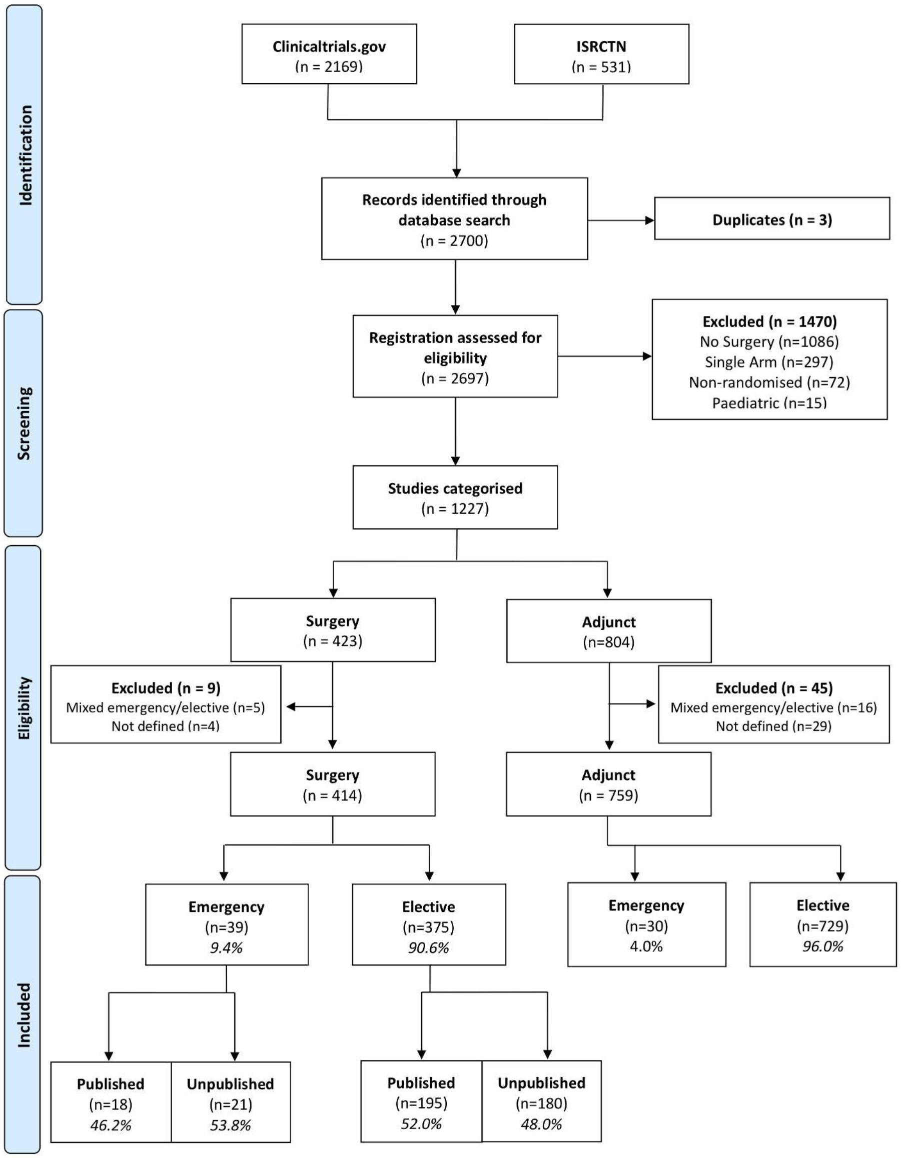

Figure 1. PRISMA style flow diagram of search results, exclusions and group numbers.

215x279mm (300 x 300 DPI)

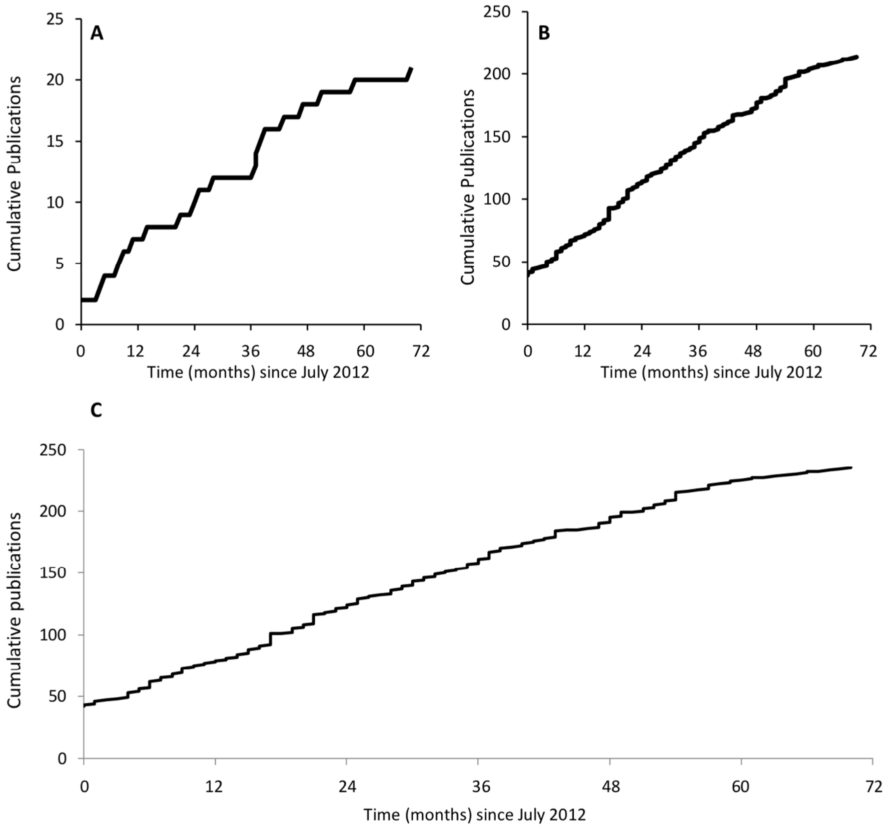

Figure 2. Kaplan-Meier curves showing publication rates of: A emergency surgical trials B elective surgical trials and C all surgical trials. Only trials published on or after the July 2012 were included, there is a cohort of trials that are registered after they started/published.

174x159mm (300 x 300 DPI)

Sponsorship	Emergency surgery; Number (% of total)	Elective surgery; Number (% of total)	All Number (% of total)
**Industry**	5 (12.8)	112 (29.9)	117 (28.6)
**Hospital**	18 (46.2)	120 (32)	138 (33.3)
**University**	10 (25.6)	92(24.5)	102 (24.6)
**Government**	5 (12.8)	21 (5.6)	26 (6.3)
**Research institute**	1 (2.6)	30 (8.0)	31 (7.5)
**TOTAL**	39 (100)	375 (100)	414 (100)

Supplementary Table 1 Comparison of the origin of sponsorship between emergency and elective surgical trials. Decimals do not add up exactly to total due to rounding.

For peer review only

BMJ Open

REGISTRATION AND PUBLICATION OF EMERGENCY AND ELECTIVE RANDOMISED CONTROLLED TRIALS IN SURGERY: A COHORT STUDY FROM TRIALS REGISTRIES

Journal:	*BMJ Open*
Manuscript ID	bmjopen-2018-021700.R2
Article Type:	Research
Date Submitted by the Author:	02-May-2018

Complete List of Authors:	Morley, Rachael Lucia; University of Bristol School of Social and Community Medicine, Centre for Surgical Research; North Bristol NHS Trust<br>Edmondson, Matthew; University of Bristol School of Social and Community Medicine, Centre for Surgical Research; Bristol Royal Infirmary<br>Rowlands, Ceri; University of Bristol School of Social and Community Medicine, Centre for Surgical Research<br>Blazeby, Jane; University of Bristol, Bristol Centre for Surgical Research, School of Social and Community Medicine; Bristol Royal Infirmary<br>Hinchliffe, Robert; University of Bristol School of Social and Community Medicine, Centre for Surgical Research; North Bristol NHS Trust

**Primary Subject Heading**:	Surgery
Secondary Subject Heading:	Emergency medicine, Health policy
Keywords:	SURGERY, EMERGENCY, HEALTH SERVICES ADMINISTRATION & MANAGEMENT

SCHOLARONE™ Manuscripts

REGISTRATION AND PUBLICATION OF EMERGENCY AND ELECTIVE RANDOMISED CONTROLLED TRIALS IN SURGERY: A COHORT STUDY FROM TRIALS REGISTRIES

Morley RL[1,2], Edmondson MJ[1,3], Rowlands C[1], Blazeby JM[1,3], Hinchliffe RJ[1,2]

[1] *Bristol Centre for Surgical Research, Bristol Medical School, University of Bristol, Bristol, UK*

[2] *North Bristol NHS Trust, Bristol, UK*

[3] *Bristol Royal Infirmary, Bristol, UK*

Corresponding author: Rachael Lucia Morley
rm17210@bristol.ac.uk
G06D, Canynge Hall, 39 Whatley Road, Bristol BS8 2PN

Key words SURGERY, EMERGENCY, HEALTH SERVICE ADMINSTRATION & MANAGEMENT

Word count 2169

For peer review only

Abstract

Word count: 291

Objectives Emergency surgical practice constitutes 50% of the workload for surgeons, but there is a lack of high quality randomised controlled trials in emergency surgery. This study aims to establish the differences between the registration, completion and publication of emergency and elective surgical trials.

Design The clinicaltrials.gov and ISRCTN.com trials registry databases were searched for randomised controlled trials between 12th July 2010 and 12th July 2012 using the keyword "surgery.' Publications were systematically searched for in Pubmed, MEDLINE and EMBASE.

Participants Results with no surgical interventions were excluded. The remaining results were manually categorised into 'emergency' or 'elective' and 'surgical' or 'adjunct' by two reviewers.

Primary outcome measures Number of randomised controlled trials (RCTs) registered in emergency versus elective surgery.

Secondary outcome measures Number of RCTs published in emergency versus elective surgery; reasons why trials remain unpublished; funding, sponsorship and impact of published articles; number of adjunct trials registered in emergency and elective surgery.

Results 2700 randomised trials were registered. 1173 trials were on a surgical population and of these, 414 trials were studying surgery. Only 9.4% (39/414) of surgical trials were in emergency surgery. The proportion of trials successfully published were similar between emergency and elective surgery (0.462 vs 0.520, mean difference -0.0584 95%CI -0.237-0.120). Unpublished emergency surgical trials were equally likely to be terminated early compared with elective trials (0.333 vs 0.156; MD -0.18, 95%CI -0.0572-0.413). Low accrual accounted for a similar majority in both groups (0.429 vs 0.464; MD-0.0357 95%CI -0.482 - 0.411). Unpublished trials in both groups were equally likely to still be planning publication (0.524 vs 0.706; MD -0.18 95%CI -0.432 - 0.0685).

Conclusion Fewer randomised controlled trials are registered in emergency than elective surgery. Once trials are registered both groups are equally likely to be published.

For peer review only

Article Summary

Strengths and limitations

- Two trials registry databases interrogated giving a large number of registrations.
- Systematic extraction of data from trials registries by two researchers.
- Publication search allowed 63 months from trial registration.
- Difficulty in categorising trials into 'emergency' or 'elective' trials based on registry data, possibility that definitions varied between trials.

Funding

This work was undertaken with the support of the MRC ConDuCT-II (Collaboration and innovation for Difficult and Complex randomised controlled Trials In Invasive procedures) Hub for Trials Methodology Research (MR/K025643/1)

This study was supported by the NIHR Biomedical Research Centre at the University Hospitals Bristol NHS Foundation Trust and the University of Bristol. The views expressed are those of the authors and not necessarily those of the UK National Health Service, National Institute for Health Research, or Department of Health.

Competing interests

The authors declare no competing interests.

Author Contribution

All authors played a substantial role in the design of the study. Morley RL collected data, synthesised results and wrote the manuscript. Edmondson MJ collected data and critically revised the manuscript. Rowlands C resolved any conflict in data collection and critically revised the manuscript. Blazeby JM critically revised the manuscript. Hinchliffe RJ resolved conflict in data collection and critically revised the manuscript. All authors gave final approval of the version to be published and agree to be held accountable for all aspects of the work.

Data Sharing Statement

Data is available upon request from the corresponding author.

For peer review only

Introduction

It is estimated that up to 50% of the workload of surgical specialties is in emergency surgical care.(1,2) In low and middle income countries, this figure rises to 60% of the workload.(3) Current evidence shows patients undergoing emergency surgery are three times more likely to die than those undergoing elective surgery. For those that survive, they are twice as likely to suffer a complication.(4,5) The UK Emergency Laparotomy Network recently revealed a variation in mortality from 3.6 to 41.7%.(6) Clearly, there is benefit to be gained from more research into emergency surgery to achieve better and more consistent outcomes.(7)

Clinical trials are essential for improving patient outcomes. A previous study has found that 52% of surgical randomised controlled trials (RCTs) remain unpublished.(8) There is currently no knowledge about the number of trials registered before publication in emergency surgery. Anecdotally, emergency surgical trials are considered more difficult to organise than their elective counterparts. Barriers to recruitment such as consent, randomisation, ethical and logistical issues can be more challenging in an emergency setting. However, it is not known whether these barriers preclude emergency surgical trials from publication any more often than elective trials.

The primary objective of this study was to determine the number of RCTs registered in emergency versus elective surgery. The secondary objectives were to compare proportions of publication in emergency versus elective surgery; reasons why trials were unpublished; variation by specialty; impact, funding and sponsorship of published trials; and the number of adjunct trials published in emergency vs elective surgery.

For peer review only

Methods

Criteria for inclusion and exclusion

Trials registered on two trials databases over a 2-year period between 12th July 2010 and 11th July 2012 that satisfied the following criteria were included; randomised controlled trials, at least one arm studying a surgical intervention or intervention within the perioperative period, adult populations, any gender from anywhere in the world. There was no limitation to the comparative group or type of outcome.

Trials with no surgery or with an intervention outside the perioperative period were excluded. Single arm and non-randomised trials were not included as they would unlikely have quality outcome data. Paediatric trials were also excluded as they are likely to have their own difficulties in trial design owing to the nature of involving children.

Definitions

Surgical interventions were defined as those involving physically changing body tissues and organs through manual operation such as cutting, suturing, abrading or the use of lasers.(9) Within this definition, this study included negative pressure wound therapy but excluded basic wound dressings (e.g self-adhesive dressing). Adjunct trials were defined as any intervention on a population of patients in the perioperative period that would not otherwise be defined as surgery e.g. anaesthesia, post-op rehabilitation, adjuvant chemotherapy.

Emergency operations were defined as unplanned admissions where it was not possible to discharge the patient home before their operation. This definition was only used when emergency or elective surgery was not specifically mentioned in the inclusion/exclusion criteria and it was not clear from the type of operation studied.

Search Strategy

Two online clinical trials databases were searched, ‘ClinicalTrials.gov,’ and the ‘International Standard Randomised Controlled Trial Number (ISRCTN) registry.’(10,11) The registry searches were performed on the same day, 31st October 2016. Search strategies for each registry respectively were as follows:

1. Keyword “surgery” for non-paediatric, phase II-IV, interventional studies registered between 12th July 2010 and 12th July 2012.

2. Keyword “surgery” for trials registered between 12th July 2010 and 12th July 2012 (paediatric trials were manually excluded)

For randomised controlled trials, the average time from registration to completion of data collection and then publication is 24 and 27 months respectively (51 months in total).(12) The 2010-2012 period was chosen to allow registered trials sufficient time to complete data collection and publication.

Two clinical trials registries were used to capture a range of trials from around the world. This aimed to avoid some selection bias if there was an unknown preference for trials to register with a specific registry.

For peer review only

Expanding the keyword search to "surgery OR operation" did not return any more results on ClinicalTrials.gov and returned disproportionally more on ISRCTN (4,290 compared to 483 for "surgery" alone). On further scrutiny, searching for "surgery OR operation" on ISRCTN database appeared to remove all other search parameters and results included studies assigned from 2000-2016. It was therefore felt 'surgery' gave the most relevant search results.

The identified RCTs were categorised (surgery vs adjunct, elective vs emergency or excluded) by two reviewers (RLM, ME). A pilot was performed where 50 titles were searched by both. Disagreements were discussed and confirmed with a senior researcher (CR and RJH). The full search was then performed with each academic trainee categorising half the trials. If registry data was unclear about the nature of operation, the publications were checked for further information. If this failed to provide adequate information, the corresponding authors were emailed (see email search). Those that could not be categorised and those including both elective and emergency patients within the same study group were excluded from the analysis. RLM and ME both also extracted data about speciality, sponsorship (industry, hospital, university, government, research institute) and funding (industry, hospital, university, government, research institute, charity or investigator).

Publication search strategy

The publication search was performed on 19th-20th October 2017, allowing a minimum 63 months from registration to publication. Trials can manually link their publication to the trials registry database and on occasion they are automatically indexed via PubMed. If this was not the case then a systematic search using Healthcare Databases Advanced Search (HDAS) was performed (selecting PubMed, Medline and EMBASE). This was repeated in Google to identify any non-indexed publications (e.g. conference abstracts). Searches were performed using; trial registration number, study title, authors, institutions and key words. Papers were matched to studies according to trial design, interventions, recruitment numbers, dates of recruitment and hypotheses.(8)

Trials were considered as published if the data appeared in journals or in full or abstract form. Published protocols or experiences from the trials were not counted. Some trials publish their data directly to the trials website and this was counted as a publication if no other publication was found.

Citation numbers were found by searching for publication title on the Web of Science Core Collection Database to indicate impact of articles. These were used as an indicator of impact of the publications.

Email search

Emails were identified from those provided on the clinical trials database. If there was no email provided, a google search for the trial investigator was performed to identify their academic or hospital email. Two standardised emails were sent. The first was sent to seek clarification of inclusion of emergency/elective patients if this remained unclear. The second email was sent to the contacts of all unpublished trials. This clarified publication status and if unpublished, a multiple-choice question of reasons why.

For peer review only

Statistical analysis

R software (R Core Team 2013. **R**: A language and environment for statistical computing. Vienna, Austria. ISBN 3-900051-07-0, URL http://www.R-project.org)(13) was used. Differences in means and the test of equal proportions were used to compare data with 95% confidence intervals.(14)

Time to publication was measured from July 2012 until month of publication. Kaplan-Meier graphs were used to visualise the rate of publication in each group.

Patient and Public Involvement

No patients were involved in the design or implementation of this study.

Results

The initial searches identified 2700 results in total. 1473 trials were initially excluded (Figure 1). Of the remaining 1227 inclusions, 423 were surgical trials and 804 were adjunct trials. Considering only surgical trials, 9 were excluded as they could not be defined as emergency or elective (n=4) or specifically included both emergency and elective patients (n=5). This gave a final total of 414 surgical trials in the analysis, of which 39 (9.4%) were emergency and 375 (90.6%) were elective trials.

Of the 414 included surgical trials, 213 (51.4%) were published. The publication of emergency surgical trials was similar to elective surgical (0.462 vs 0.520, mean difference -0.0584 95%CI -0.237-0.120). The proportions of publications over time in each group are shown in figure 2, demonstratinga relative plateau at 55 months. There was variation across specialties in the number of emergency and elective surgical trials registered and published (Table 1). Citation numbers were similar in emergency (mean 21.75, range 0-98) and elective (mean 21.59, range 0-306) surgical trials (difference 0.16 95%CI -16.7 to 16.9).

Speciality		Trials registered; Number (% of trials in specialty)	Trials published; Number (% of trials registered)
General Surgery (All)	Elective	111 (84.7)	59 (53.2)
	Emergency	20 (15.3)	13 (65.0)
Orthopaedics	Elective	86 (86.9)	38 (44.2)
	Emergency	13 (13.1)	3 (23.1)
General (Lower GI)*	Elective	43 (84.3)	27 (62.8)
	Emergency	8 (15.7)	6 (75.0)
General (Upper GI)*	Elective	41 (89.1)	17 (41.5)
	Emergency	5 (10.9)	3 (60.0)
Ophthalmology	Elective	34 (100)	22 (64.7)
	Emergency	0 (0)	-
Cardiothoracic	Elective	33 (97.1)	18 (64.3)
	Emergency	1 (2.9)	0 (0)
Obstetrics &	Elective	29 (96.7)	13 (44.8)

For peer review only

**Gynaecology**	Emergency	1 (3.3)	0 (0)
**Neurosurgery**	Elective	24 (92.3)	8 (33.3)
	Emergency	2 (7.7)	2 (100)
**Vascular**	Elective	18 (100)	11 (61.1)
	Emergency	0 (0)	-
**General (Other)***	Elective	10 (58.2)	7 (70.0)
	Emergency	7 (41.8)	4 (57.1)
**General (Breast)***	Elective	14 (100)	6 (42.9)
	Emergency	0 (0)	-
**Urology**	Elective	14 (100)	7 (50.0)
	Emergency	0 (0)	-
**Plastics**	Elective	11 (84.6)	8 (72.7)
	Emergency	2 (15.4)	0 (0)
**Other**	Elective	15 (100)	11 (73.3)
	Emergency	0 (0)	-
**TOTAL**	Elective	375 (100)	195 (52.0)
	Emergency	39 (100)	18 (46.2)

*Table 1 Surgical trials by speciality (excluding specialities with less than ten trials found) showing proportion of elective vs emergency trials and the percentage of these that were published. * Categories combine into general surgery (all). Only general surgery (all) was included in the totals*

Most publications were identified from citations included within trial registrations (n=110). A substantial proportion were identified by searching HDAS and Google (n=90). Few publications were found via email responses (n=4) or data published only on trial registrations (n=9).

In searching for publications and clarifications of definitions, 148 emails were sent. Outcomes from emails sent were; no response (n=108), undeliverable (n=13) or replied (n=27). Many trials had no contact available (n=69).

Of the 201 unpublished trials, 21 (10.4%) were emergency and 180 (89.6%) were elective surgery. Reasons for non-publication of emergency and elective trials respectively were; completed but awaiting data analysis/publication process (7/21, 33.3%; 62/180, 34.4%), ongoing data collection (4/21, 19.0%; 65/180, 36.1%), terminated trial (7/21, 33.3%; 28/180, 15.6%) and unknown (3/21, 14.3%; 25/180, 13.9%;).

Unpublished trials in both groups were equally likely to still be planning publication (awaiting data analysis or ongoing data collection; 0.524 vs 0.706; MD -0.18 95%CI -0.432 -0.0685; table 2). Unpublished emergency surgical trials were equally likely to be terminated early compared with elective trials (0.333 vs 0.156; MD -0.18, 95%CI -0.0572-0.413). Low accrual accounted for a similar majority in both groups (0.429 vs 0.464; MD-0.0357 95%CI -0.482 -0.411) .

For peer review only

	Emergency surgery; Number (% of total)	Elective surgery; Number (% of total)	All Number (%)
**COMPLETED AND AWAITING PUBLICATION**	7 (33.3)	62 (34.4)	69 (34.3)
**ONGOING DATA COLLECTION**	4 (19.0)	65 (36.1)	69 (34.3)
**TERMINATED**	7 (33.3)	29 (15.6)	36 (17.9)
**UNKNOWN**	3 (14.3)	24 (13.9)	27 (13.4)
**TOTAL**	21 (100)	180 (100)	201 (100)

Table 2 Reasons for trials remaining unpublished. Decimals do not add up exactly to total due to rounding.

Funding categories were only listed on ISRCTN and not clinicaltrials.gov. Not all trials registered listed sponsorship. Most trials were sponsored by hospitals. Of published trials, there were no significant differences in sponsorship (supplementary table 1). Most surgical trials were funded by government bodies (table 3).

FUNDING	EMERGENCY SURGERY; NUMBER (% OF TOTAL)	ELECTIVE SURGERY; NUMBER (% OF TOTAL)	ALL NUMBER (%)
**INDUSTRY**	0 (0)	15 (18.1)	15 (16.5)
**HOSPITAL**	2 (25.0)	18 (21.7)	20 (22.0)
**UNIVERSITY**	0 (0)	13 (15.7)	13 (14.3)
**GOVERNMENT**	5 (62.5)	18 (21.7)	23 (25.3)
**RESEARCH INSTITUTE**	1 (12.5)	7 (8.4)	8 (8.8)
**CHARITY**	0 (0)	9 (10.8)	9 (10.0)
**INVESTIGATOR**	0 (0)	3 (3.6)	3 (3.3)
**TOTAL**	8 (100)	83 (100)	91 (100)

Table 3 Comparison of the origin of funding between emergency and elective surgical trials. This data was only available from ISRCTN and not clinicaltrials.gov. Decimals do not add up exactly to total due to rounding.

Of 804 adjunct trials registered, 45 were excluded as they could not be defined (n=29) or they included both elective and emergency patients (n=16). This left 759 adjunct trials that

For peer review only

were analysed. Elective trials represented 96% (n=729) whilst emergency trials only contributed 4% (n=30). This paper did not consider the non-publication of adjunct trials.

Discussion

This study found that there are significantly fewer randomised controlled trials registered in emergency surgery. However, emergency surgical trials that were registered were equally as likely to be published as elective surgical trials. Recruitment difficulties leading to early trial termination were encountered at a similar rate between elective and emergency studies. Similarly, there was no clear difference between citation numbers in emergency and elective trials, which was used as a marker for research impact. Variation between specialties revealed a comparable pattern to the overall picture.

Registration and publication of all surgical trials has previously been studied.(8) Emergency and elective trials were not studied separately, but found that 48% of all surgical trials remained published at 38 months. The current paper allowed a further 25 months minimum for our publication search and the overall publication proportion was similar (51.5%). This suggests a relative plateau in the rate of publication of the trials beyond 38 months. Interpretation of figure 2 however sees a plateau at around 55 months. Whilst more publications could be expected after this time, extending the search interval may limit applicability to current practice.

The discontinuation of RCTs in critical care has also been studied, which included seven surgical trials.(15) They found the percentage of 'acute care' trials (critical care or care within 24 hours of presentation) was 7%, and a similar publication rate between 'acute' and 'non-acute' trials. However, slow recruitment caused twice as many acute trials to be discontinued than non-acute trials. The methodology was based on comparing published protocols and publications, as opposed to trial registration and publications.

This study has several limitations. Emergency was a binary definition reflecting the wording used within the trials registries. There are inherent difficulties when trying to categorise patients this way, as some operations are 'urgent' but not 'emergency' (e.g. bone fractures). It is likely that within the trials searched, the definition of emergency varied. It is possible that this paper gives an overestimation of true emergency surgical trials, where 'urgent' trials have been upgraded to 'emergency' in attempting to define them.

Although this paper demonstrates a lack of registration of emergency surgical trials compared to surgical workload, further work is required to elicit the reasons. Because emergency trials are traditionally perceived as more difficult to achieve, both clinicians and sponsors may be more hesitant to invest their resources. This study shows that emergency surgical trials are equally as likely to be published as elective surgical trials and are a similar investment risk to elective randomised controlled trials. Indeed, many issues around consent and randomisation in emergency situations are being overcome.(16–20) With this knowledge, clinicians, researchers and sponsors may feel reassured and inclined to be involved with emergency surgical trials.

For peer review only - http://bmjopen.bmj.com/site/about/guidelines.xhtml

Emergency surgery is under-represented in the literature, despite the high volume of workload it creates and poor outcomes. This study shows that registered emergency and elective surgical trials are equally as likely to be published with comparable recruitment issues. A perceived lack of successful completion and reporting of emergency surgical trials should no longer prevent investment into studies that may positively impact emergency surgery.

For peer review only

Figure Legend

Figure 1. PRISMA style flow diagram of search results, exclusions and group numbers.

Figure 2. Kaplan-Meier curves showing publication over time of emergency (blue) and elective (green) surgical trials. Only trials published on or after the July 2012 were included, there is a cohort of trials that are registered after they started/published.

For peer review only

References

1. Gale SC, Shafi S, Dombrovskiy VY, Arumugam D, Crystal JS. The public health burden of emergency general surgery in the United States. J Trauma Acute Care Surg [Internet]. 2014 Aug [cited 2017 Aug 3];77(2):202–8. Available from: http://www.ncbi.nlm.nih.gov/pubmed/25058242

2. RCSEng. Emergency Surgery - Standards for unscheduled surgical care [Internet]. 2011 [cited 2017 Oct 5]. Available from: https://www.google.co.uk/url?sa=t&rct=j&q=&esrc=s&source=web&cd=3&cad=rja&uact=8&ved=0ahUKEwiltJHc-JfXAhXBHxoKHcrzC7EQFggyMAI&url=https%3A%2F%2Fwww.rcseng.ac.uk%2F-%2Fmedia%2Ffiles%2Frcs%2Fabout-rcs%2Fregional%2Frcs_emergency_surgery_2011_web.pdf&usg=AOvV

3. McCord C, Ozgediz D, Beard JH, Debas HT. General Surgical Emergencies [Internet]. Essential Surgery: Disease Control Priorities, Third Edition (Volume 1). The International Bank for Reconstruction and Development / The World Bank; 2015 [cited 2017 Jul 3]. Available from: http://www.ncbi.nlm.nih.gov/pubmed/26741004

4. Mullen MG, Michaels AD, Mehaffey JH, Guidry CA, Turrentine FE, Hedrick TL, et al. Risk Associated With Complications and Mortality After Urgent Surgery vs Elective and Emergency Surgery. JAMA Surg [Internet]. 2017 May 10 [cited 2017 Jul 3]; Available from: http://www.ncbi.nlm.nih.gov/pubmed/28492821

5. Visser A, Geboers B, Gouma DJ, Goslings JC, Ubbink DT. Predictors of surgical complications: A?systematic review. Surgery [Internet]. 2015 Jul [cited 2017 Jul 5];158(1):58–65. Available from: http://linkinghub.elsevier.com/retrieve/pii/S0039606015000288

6. Saunders DI, Murray D, Pichel AC, Varley S, Peden CJ, UK Emergency Laparotomy Network. Variations in mortality after emergency laparotomy: the first report of the UK Emergency Laparotomy Network. Br J Anaesth [Internet]. 2012 Sep 1 [cited 2017 Nov 13];109(3):368–75. Available from: http://www.ncbi.nlm.nih.gov/pubmed/22728205

7. Becher RD, Hoth JJ, Miller PR, Mowery NT, Chang MC, Meredith JW. A critical assessment of outcomes in emergency versus nonemergency general surgery using the American College of Surgeons National Surgical Quality Improvement Program database. Am Surg [Internet]. 2011 Jul [cited 2017 Oct 5];77(7):951–9. Available from: http://www.ncbi.nlm.nih.gov/pubmed/21944366

8. Chapman SJ, Shelton B, Mahmood H, Fitzgerald JE, Harrison EM, Bhangu A. Discontinuation and non-publication of surgical randomised controlled trials: observational study. BMJ [Internet]. 2014 Dec 9 [cited 2016 Oct 31];349(dec09_1):g6870. Available from: http://www.bmj.com/content/349/bmj.g6870

For peer review only

9. Cook JA. The challenges faced in the design, conduct and analysis of surgical randomised controlled trials. Trials [Internet]. 2009 Dec 6 [cited 2017 Oct 19];10(1):9. Available from: http://www.ncbi.nlm.nih.gov/pubmed/19200379

10. BioMed Central Ltd. ISRCTN Registry [Internet]. [cited 2016 Oct 31]. Available from: http://www.isrctn.com/

11. U.S. National Library of Medicine. ClinicalTrials.gov [Internet]. [cited 2016 Oct 31]. Available from: https://clinicaltrials.gov/

12. Walker KF, Stevenson G, Thornton JG. Discrepancies between registration and publication of randomised controlled trials: an observational study. JRSM open [Internet]. SAGE Publications; 2014 May 1 [cited 2016 Oct 31];5(5):2042533313517688. Available from: http://shr.sagepub.com/content/5/5/2042533313517688.full

13. R Development Core Team (2008). A language and environment for statistical computing. R Foundation for Statistical Computing, Vienna, Austria [Internet]. p. http://www.r-project.org [accessed 13th Nov 2017]. Available from: http://www.r-project.org

14. Newcombe RG. TWO-SIDED CONFIDENCE INTERVALS FOR THE SINGLE PROPORTION: COMPARISON OF SEVEN METHODS. Stat Med Stat Med [Internet]. 1998 [cited 2018 Apr 23];17:857–72. Available from: http://citeseerx.ist.psu.edu/viewdoc/download?doi=10.1.1.408.7107&rep=rep1&type=pdf

15. Schandelmaier S, von Elm E, You JJ, Blümle A, Tomonaga Y, Lamontagne F, et al. Premature Discontinuation of Randomized Trials in Critical and Emergency Care. Crit Care Med [Internet]. 2016 Jan [cited 2017 Nov 13];44(1):130–7. Available from: http://www.ncbi.nlm.nih.gov/pubmed/26468895

16. Edwards M, Grocott M, Pearse R. Study Documents - FLuid Optimisation in Emergency LAparotomy trial [Internet]. 2017 [cited 2017 Nov 13]. Available from: http://www.floela.org/documents

17. Jansen J, Campbell M. UK-REBOA [Internet]. 2017 [cited 2017 Nov 13]. Available from: https://w3.abdn.ac.uk/hsru/REBOA/Public/Public/index.cshtml

18. Taylor J, Black S, J. Brett S, Kirby K, Nolan JP, Reeves BC, et al. Design and implementation of the AIRWAYS-2 trial: A multi-centre cluster randomised controlled trial of the clinical and cost effectiveness of the i-gel supraglottic airway device versus tracheal intubation in the initial airway management of out of hospital cardiac arrest. Resuscitation [Internet]. 2016 Dec [cited 2017 Nov 13];109:25–32. Available from: http://www.ncbi.nlm.nih.gov/pubmed/27697605

19. Roberts I, Shakur H, Coats T, Hunt B, Balogun E, Barnetson L, et al. The CRASH-2 trial: a randomised controlled trial and economic evaluation of the effects of tranexamic

For peer review only

acid on death, vascular occlusive events and transfusion requirement in bleeding trauma patients. Heal Technol Assess [Internet]. 2013 Mar [cited 2017 Aug 3];17(10):1–79. Available from: http://www.ncbi.nlm.nih.gov/pubmed/23477634

20. IMPROVE Trial Investigators I trial, Powell JT, Sweeting MJ, Thompson MM, Ashleigh R, Bell R, et al. Endovascular or open repair strategy for ruptured abdominal aortic aneurysm: 30 day outcomes from IMPROVE randomised trial. BMJ [Internet]. British Medical Journal Publishing Group; 2014 Jan 13 [cited 2017 Nov 13];348:f7661. Available from: http://www.ncbi.nlm.nih.gov/pubmed/24418950

For peer review only

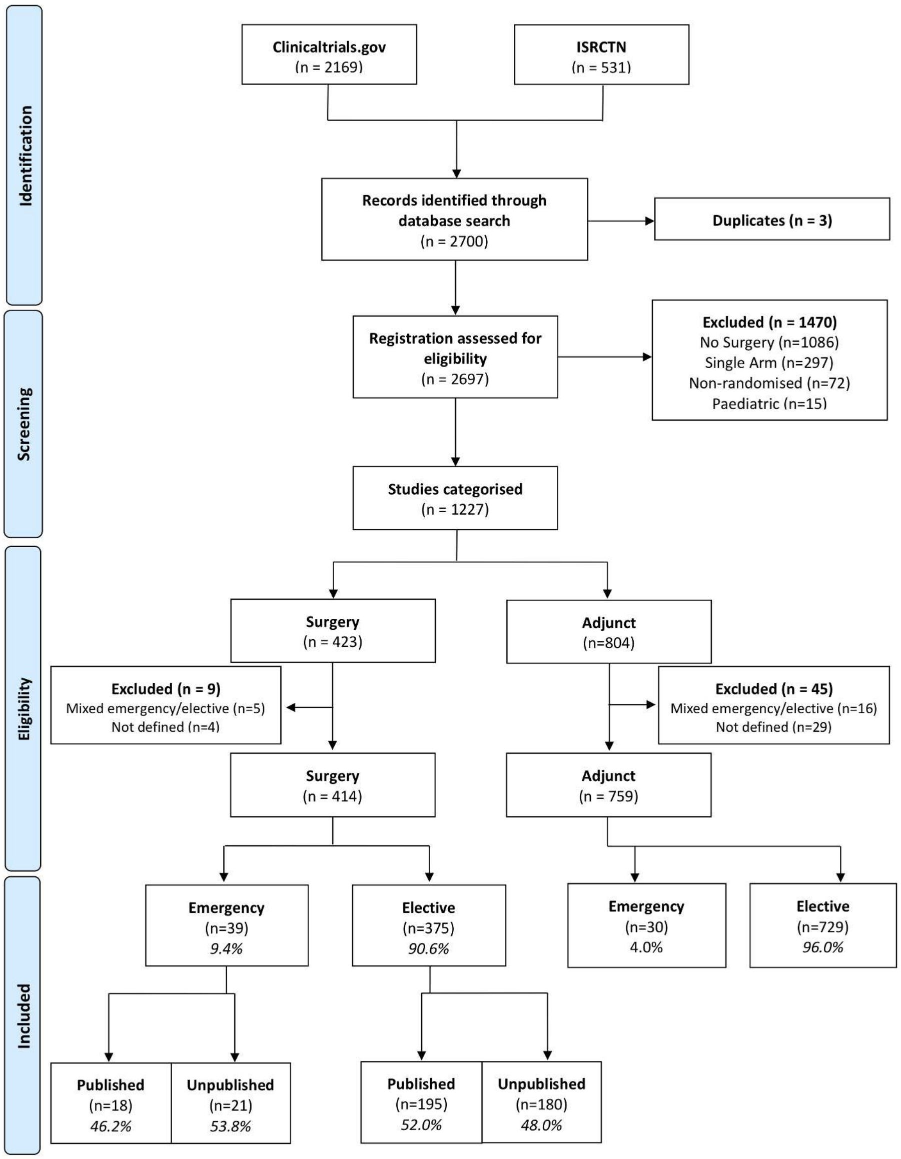

Figure 1. PRISMA style flow diagram of search results, exclusions and group numbers.

215x279mm (300 x 300 DPI)

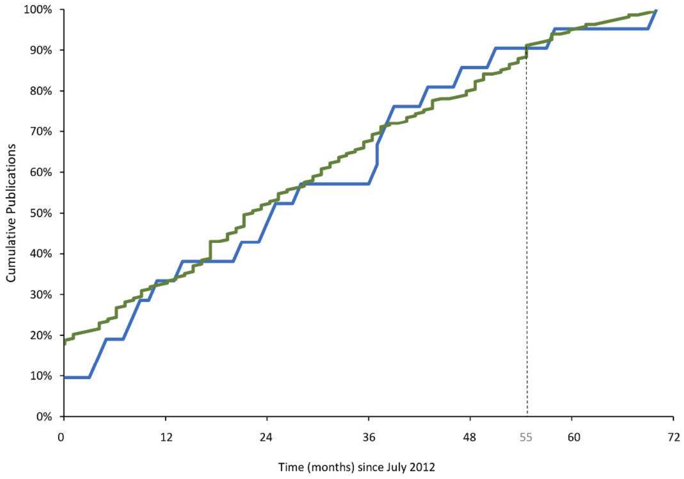

Kaplan-Meier curves showing publication over time of emergency (blue) and elective (green) surgical trials. Only trials published on or after the July 2012 were included, there is a cohort of trials that are registered after they started/published.

209x297mm (300 x 300 DPI)

Sponsorship	Emergency surgery; Number (% of total)	Elective surgery; Number (% of total)	All Number (% of total)
**Industry**	5 (12.8)	112 (29.9)	117 (28.6)
**Hospital**	18 (46.2)	120 (32)	138 (33.3)
**University**	10 (25.6)	92(24.5)	102 (24.6)
**Government**	5 (12.8)	21 (5.6)	26 (6.3)
**Research institute**	1 (2.6)	30 (8.0)	31 (7.5)
**TOTAL**	39 (100)	375 (100)	414 (100)

Supplementary Table 1 Comparison of the origin of sponsorship between emergency and elective surgical trials. Decimals do not add up exactly to total due to rounding.

For peer review only

BMJ Open

REGISTRATION AND PUBLICATION OF EMERGENCY AND ELECTIVE RANDOMISED CONTROLLED TRIALS IN SURGERY: A COHORT STUDY FROM TRIALS REGISTRIES

Journal:	*BMJ Open*
Manuscript ID	bmjopen-2018-021700.R3
Article Type:	Research
Date Submitted by the Author:	24-May-2018

Complete List of Authors:	Morley, Rachael Lucia; University of Bristol School of Social and Community Medicine, Centre for Surgical Research; North Bristol NHS Trust<br>Edmondson, Matthew; University of Bristol School of Social and Community Medicine, Centre for Surgical Research; Bristol Royal Infirmary<br>Rowlands, Ceri; University of Bristol School of Social and Community Medicine, Centre for Surgical Research<br>Blazeby, Jane; University of Bristol, Bristol Centre for Surgical Research, School of Social and Community Medicine; Bristol Royal Infirmary<br>Hinchliffe, Robert; University of Bristol School of Social and Community Medicine, Centre for Surgical Research; North Bristol NHS Trust

**Primary Subject Heading**:	Surgery
Secondary Subject Heading:	Emergency medicine, Health policy
Keywords:	SURGERY, EMERGENCY, HEALTH SERVICES ADMINISTRATION & MANAGEMENT

SCHOLARONE™ Manuscripts

REGISTRATION AND PUBLICATION OF EMERGENCY AND ELECTIVE RANDOMISED CONTROLLED TRIALS IN SURGERY: A COHORT STUDY FROM TRIALS REGISTRIES

Morley RL[1,2], Edmondson MJ[1,3], Rowlands C[1], Blazeby JM[1,3], Hinchliffe RJ[1,2]

[1] *Bristol Centre for Surgical Research, Bristol Medical School, University of Bristol, Bristol, UK*

[2] *North Bristol NHS Trust, Bristol, UK*

[3] *Bristol Royal Infirmary, Bristol, UK*

Corresponding author: Rachael Lucia Morley
rm17210@bristol.ac.uk
G06D, Canynge Hall, 39 Whatley Road, Bristol BS8 2PN

Key words SURGERY, EMERGENCY, HEALTH SERVICE ADMINSTRATION & MANAGEMENT

Word count 2210

For peer review only

Abstract

Word count: 295

Objectives Emergency surgical practice constitutes 50% of the workload for surgeons, but there is a lack of high quality randomised controlled trials in emergency surgery. This study aims to establish the differences between the registration, completion and publication of emergency and elective surgical trials.

Design The clinicaltrials.gov and ISRCTN.com trials registry databases were searched for randomised controlled trials between 12th July 2010 and 12th July 2012 using the keyword "surgery.' Publications were systematically searched for in Pubmed, MEDLINE and EMBASE.

Participants Results with no surgical interventions were excluded. The remaining results were manually categorised into 'emergency' or 'elective' and 'surgical' or 'adjunct' by two reviewers.

Primary outcome measures Number of randomised controlled trials (RCTs) registered in emergency versus elective surgery.

Secondary outcome measures Number of RCTs published in emergency versus elective surgery; reasons why trials remain unpublished; funding, sponsorship and impact of published articles; number of adjunct trials registered in emergency and elective surgery.

Results 2700 randomised trials were registered. 1173 trials were on a surgical population and of these, 414 trials were studying surgery. Only 9.4% (39/414) of surgical trials were in emergency surgery. The proportion of trials successfully published did not significantly differ between emergency and elective surgery (0.46 vs 0.52, mean difference -0.06 95%CI -0.24-0.12). Unpublished emergency surgical trials were statistically equally likely to be terminated early compared with elective trials (0.33 vs 0.16; MD -0.18, 95%CI -0.06-0.41). Low accrual accounted for a similar majority in both groups (0.43 vs 0.46; MD-0.04 95%CI -0.48 -0.41). Unpublished trials in both groups were statistically equally likely to still be planning publication (0.52 vs 0.71; MD -0.18 95%CI -0.43 - 0.07).

Conclusion Fewer randomised controlled trials are registered in emergency than elective surgery. Once trials are registered both groups are equally likely to be published.

For peer review only - http://bmjopen.bmj.com/site/about/guidelines.xhtml

Article Summary

Strengths and limitations

- Two trials registry databases interrogated giving a large number of registrations.
- Systematic extraction of data from trials registries by two researchers.
- Publication search allowed 63 months from trial registration.
- Difficulty in categorising trials into 'emergency' or 'elective' trials based on registry data, possibility that definitions varied between trials.

Funding

This work was undertaken with the support of the MRC ConDuCT-II (Collaboration and innovation for Difficult and Complex randomised controlled Trials In Invasive procedures) Hub for Trials Methodology Research (MR/K025643/1)

This study was supported by the NIHR Biomedical Research Centre at the University Hospitals Bristol NHS Foundation Trust and the University of Bristol. The views expressed are those of the authors and not necessarily those of the UK National Health Service, National Institute for Health Research, or Department of Health.

Competing interests

The authors declare no competing interests.

Author Contribution

All authors played a substantial role in the design of the study. Morley RL collected data, synthesised results and wrote the manuscript. Edmondson MJ collected data and critically revised the manuscript. Rowlands C resolved any conflict in data collection and critically revised the manuscript. Blazeby JM critically revised the manuscript. Hinchliffe RJ resolved conflict in data collection and critically revised the manuscript. All authors gave final approval of the version to be published and agree to be held accountable for all aspects of the work.

Data Sharing Statement

Data is available upon request from the corresponding author.

For peer review only

Introduction

It is estimated that up to 50% of the workload of surgical specialties is in emergency surgical care.(1,2) In low and middle income countries, this figure rises to 60% of the workload.(3) Current evidence shows patients undergoing emergency surgery are three times more likely to die than those undergoing elective surgery. For those that survive, they are twice as likely to suffer a complication.(4,5) The UK Emergency Laparotomy Network recently revealed a variation in mortality from 3.6 to 41.7%.(6) Clearly, there is benefit to be gained from more research into emergency surgery to achieve better and more consistent outcomes.(7)

Clinical trials are essential for improving patient outcomes. A previous study has found that 52% of surgical randomised controlled trials (RCTs) remain unpublished.(8) There is currently no knowledge about the number of trials registered before publication in emergency surgery. Anecdotally, emergency surgical trials are considered more difficult to organise than their elective counterparts. Barriers to recruitment such as consent, randomisation, ethical and logistical issues can be more challenging in an emergency setting. However, it is not known whether these barriers preclude emergency surgical trials from publication any more often than elective trials.

The primary objective of this study was to determine the number of RCTs registered in emergency versus elective surgery. The secondary objectives were to compare proportions of publication in emergency versus elective surgery; reasons why trials were unpublished; variation by specialty; impact, funding and sponsorship of published trials; and the number of adjunct trials published in emergency vs elective surgery.

For peer review only

Methods

Criteria for inclusion and exclusion

Trials registered on two trials databases over a 2-year period between 12th July 2010 and 11th July 2012 that satisfied the following criteria were included; randomised controlled trials, at least one arm studying a surgical intervention or intervention within the perioperative period, adult populations, any gender from anywhere in the world. There was no limitation to the comparative group or type of outcome.

Trials with no surgery or with an intervention outside the perioperative period were excluded. Single arm and non-randomised trials were not included as they would unlikely have quality outcome data. Paediatric trials were also excluded as they are likely to have their own difficulties in trial design owing to the nature of involving children.

Definitions

Surgical interventions were defined as those involving physically changing body tissues and organs through manual operation such as cutting, suturing, abrading or the use of lasers.(9) Within this definition, this study included negative pressure wound therapy but excluded basic wound dressings (e.g self-adhesive dressing). Adjunct trials were defined as any intervention on a population of patients in the perioperative period that would not otherwise be defined as surgery e.g. anaesthesia, post-op rehabilitation, adjuvant chemotherapy.

Emergency operations were defined as unplanned admissions where it was not possible to discharge the patient home before their operation. This definition was only used when emergency or elective surgery was not specifically mentioned in the inclusion/exclusion criteria and it was not clear from the type of operation studied.

Search Strategy

Two online clinical trials databases were searched, 'ClinicalTrials.gov,' and the 'International Standard Randomised Controlled Trial Number (ISRCTN) registry.'(10,11) The registry searches were performed on the same day, 31st October 2016. Search strategies for each registry respectively were as follows:

1. Keyword "surgery" for non-paediatric, phase II-IV, interventional studies registered between 12th July 2010 and 12th July 2012.

2. Keyword "surgery" for trials registered between 12th July 2010 and 12th July 2012 (paediatric trials were manually excluded)

For randomised controlled trials, the average time from registration to completion of data collection and then publication is 24 and 27 months respectively (51 months in total).(12) The 2010-2012 period was chosen to allow registered trials sufficient time to complete data collection and publication.

Two clinical trials registries were used to capture a range of trials from around the world. This aimed to avoid some selection bias if there was an unknown preference for trials to register with a specific registry.

For peer review only

Expanding the keyword search to "surgery OR operation" did not return any more results on ClinicalTrials.gov and returned disproportionally more on ISRCTN (4,290 compared to 483 for "surgery" alone). On further scrutiny, searching for "surgery OR operation" on ISRCTN database appeared to remove all other search parameters and results included studies assigned from 2000-2016. It was therefore felt 'surgery' gave the most relevant search results.

The identified RCTs were categorised (surgery vs adjunct, elective vs emergency or excluded) by two reviewers (RLM, ME). A pilot was performed where 50 titles were searched by both. Disagreements were discussed and confirmed with a senior researcher (CR and RJH). The full search was then performed with each academic trainee categorising half the trials. If registry data was unclear about the nature of operation, the publications were checked for further information. If this failed to provide adequate information, the corresponding authors were emailed (see email search). Those that could not be categorised and those including both elective and emergency patients within the same study group were excluded from the analysis. RLM and ME both also extracted data about speciality, sponsorship (industry, hospital, university, government, research institute) and funding (industry, hospital, university, government, research institute, charity or investigator).

Publication search strategy

The publication search was performed on 19th-20th October 2017, allowing a minimum 63 months from registration to publication. Trials can manually link their publication to the trials registry database and on occasion they are automatically indexed via PubMed. If this was not the case then a systematic search using Healthcare Databases Advanced Search (HDAS) was performed (selecting PubMed, Medline and EMBASE). This was repeated in Google to identify any non-indexed publications (e.g. conference abstracts). Searches were performed using; trial registration number, study title, authors, institutions and key words. Papers were matched to studies according to trial design, interventions, recruitment numbers, dates of recruitment and hypotheses.(8)

Trials were considered as published if the data appeared in journals or in full or abstract form. Published protocols or experiences from the trials were not counted. Some trials publish their data directly to the trials website and this was counted as a publication if no other publication was found.

Citation numbers were found by searching for publication title on the Web of Science Core Collection Database to indicate impact of articles. These were used as an indicator of impact of the publications.

Email search

Emails were identified from those provided on the clinical trials database. If there was no email provided, a google search for the trial investigator was performed to identify their academic or hospital email. Two standardised emails were sent. The first was sent to seek clarification of inclusion of emergency/elective patients if this remained unclear. The second email was sent to the contacts of all unpublished trials. This clarified publication status and if unpublished, a multiple-choice question of reasons why.

For peer review only

Statistical analysis

R software (R Core Team 2013. **R**: A language and environment for statistical computing. Vienna, Austria. ISBN 3-900051-07-0, URL http://www.R-project.org)(13) was used. Differences in means and the test of equal proportions were used to compare data with 95% confidence intervals.(14)

Time to publication was measured from July 2012 until month of publication. Kaplan-Meier graphs were used to visualise the rate of publication in each group.

Patient and Public Involvement

No patients were involved in the design or implementation of this study.

For peer review only

Results

The initial searches identified 2700 results in total. 1473 trials were initially excluded (Figure 1). Of the remaining 1227 inclusions, 423 were surgical trials and 804 were adjunct trials. Considering only surgical trials, 9 were excluded as they could not be defined as emergency or elective (n=4) or specifically included both emergency and elective patients (n=5). This gave a final total of 414 surgical trials in the analysis, of which 39 (9.4%) were emergency and 375 (90.6%) were elective trials.

Surgical Trials

Of the 414 included surgical trials, 213 (51.4%) were published. The publication of emergency surgical trials was not statistically different to elective surgical (0.46 vs 0.52, mean difference -0.06 95%CI -0.24-0.12). The proportions of publications over time in each group are shown in figure 2, demonstrating a relative plateau at 55 months. There was variation across specialties in the number of emergency and elective surgical trials registered and published (Table 1). The number of citations were similar in emergency (mean 21.75, range 0-98) and elective (mean 21.59, range 0-306) surgical trials (difference 0.16 95%CI -16.7 to 16.9).

Speciality		Trials registered; Number (% of trials in specialty)	Trials published; Number (% of trials registered)
**General Surgery (All)**	Elective	111 (84.7)	59 (53.2)
	Emergency	20 (15.3)	13 (65.0)
**Orthopaedics**	Elective	86 (86.9)	38 (44.2)
	Emergency	13 (13.1)	3 (23.1)
**General (Lower GI)***	Elective	43 (84.3)	27 (62.8)
	Emergency	8 (15.7)	6 (75.0)
**General (Upper GI)***	Elective	41 (89.1)	17 (41.5)
	Emergency	5 (10.9)	3 (60.0)
**Ophthalmology**	Elective	34 (100)	22 (64.7)
	Emergency	0 (0)	-
**Cardiothoracic**	Elective	33 (97.1)	18 (64.3)

	Emergency	1 (2.9)	0 (0)
**Obstetrics & Gynaecology**	Elective	29 (96.7)	13 (44.8)
	Emergency	1 (3.3)	0 (0)
**Neurosurgery**	Elective	24 (92.3)	8 (33.3)
	Emergency	2 (7.7)	2 (100)
**Vascular**	Elective	18 (100)	11 (61.1)
	Emergency	0 (0)	-
**General (Other)***	Elective	10 (58.2)	7 (70.0)
	Emergency	7 (41.8)	4 (57.1)
**General (Breast)***	Elective	14 (100)	6 (42.9)
	Emergency	0 (0)	-
**Urology**	Elective	14 (100)	7 (50.0)
	Emergency	0 (0)	-
**Plastics**	Elective	11 (84.6)	8 (72.7)
	Emergency	2 (15.4)	0 (0)
**Other**	Elective	15 (100)	11 (73.3)
	Emergency	0 (0)	-
**TOTAL**	Elective	375 (100)	195 (52.0)
	Emergency	39 (100)	18 (46.2)

*Table 1 Surgical trials by speciality (excluding specialities with less than ten trials found) showing proportion of elective vs emergency trials and the percentage of these that were published. * Categories combine into general surgery (all). Only general surgery (all) was included in the totals*

Most publications were identified from citations included within trial registrations (n=110). A substantial proportion were identified by searching HDAS and Google (n=90). Few publications were found via email responses (n=4) or data published only on trial registrations (n=9).

In searching for publications and clarifications of definitions, 148 emails were sent. Outcomes from emails sent were; no response (n=108), undeliverable (n=13) or replied (n=27). Many trials had no contact available (n=69).

Of the 201 unpublished trials, 21 (10.4%) were emergency and 180 (89.6%) were elective surgery. Reasons for non-publication of emergency and elective trials respectively were; completed but awaiting data analysis/publication process (7/21, 33.3%; 62/180, 34.4%), ongoing data collection (4/21, 19.0%; 65/180, 36.1%), terminated trial (7/21, 33.3%; 28/180, 15.6%) and unknown (3/21, 14.3%; 25/180, 13.9%;).

Unpublished trials in both groups were statistically equally likely to still be planning publication (awaiting data analysis or ongoing data collection; 0.52 vs 0.71; MD -0.18 95%CI -0.43 - 0.07; table 2). Unpublished emergency surgical trials were statistically equally likely to be terminated early compared with elective trials (0.33 vs 0.16; MD -0.18, 95%CI -0.06-0.41). Low accrual accounted for a statistically similar majority in both groups (0.43 vs 0.46; MD-0.04 95%CI -0.48 -0.41).

For peer review only

	Emergency surgery; Number (% of total)	Elective surgery; Number (% of total)	All Number (%)
COMPLETED AND AWAITING PUBLICATION	7 (33.3)	62 (34.4)	69 (34.3)
ONGOING DATA COLLECTION	4 (19.0)	65 (36.1)	69 (34.3)
TERMINATED	7 (33.3)	29 (15.6)	36 (17.9)
UNKNOWN	3 (14.3)	24 (13.9)	27 (13.4)
TOTAL	21 (100)	180 (100)	201 (100)

Table 2 Reasons for trials remaining unpublished. Decimals do not add up exactly to total due to rounding.

Funding categories were only listed on ISRCTN and not clinicaltrials.gov. Not all trials registered listed sponsorship. Most trials were sponsored by hospitals. Of published trials, there were no significant differences in sponsorship (supplementary table 1). Most surgical trials were funded by government bodies (table 3).

FUNDING	EMERGENCY SURGERY; NUMBER (% OF TOTAL)	ELECTIVE SURGERY; NUMBER (% OF TOTAL)	ALL NUMBER (%)
INDUSTRY	0 (0)	15 (18.1)	15 (16.5)
HOSPITAL	2 (25.0)	18 (21.7)	20 (22.0)
UNIVERSITY	0 (0)	13 (15.7)	13 (14.3)
GOVERNMENT	5 (62.5)	18 (21.7)	23 (25.3)
RESEARCH INSTITUTE	1 (12.5)	7 (8.4)	8 (8.8)
CHARITY	0 (0)	9 (10.8)	9 (10.0)
INVESTIGATOR	0 (0)	3 (3.6)	3 (3.3)
TOTAL	8 (100)	83 (100)	91 (100)

Table 3 Comparison of the origin of funding between emergency and elective surgical trials. This data was only available from ISRCTN and not clinicaltrials.gov. Decimals do not add up exactly to total due to rounding.

For peer review only

Adjunct Trials

Of 804 adjunct trials registered, 45 were excluded as they could not be defined (n=29) or they included both elective and emergency patients (n=16). This left 759 adjunct trials that were analysed. Elective trials represented 96% (n=729) whilst emergency trials only contributed 4% (n=30). This paper did not consider the non-publication of adjunct trials.

Discussion

This study found that there are significantly fewer randomised controlled trials registered in emergency surgery. However, emergency surgical trials that were registered were equally as likely to be published as elective surgical trials. Recruitment difficulties leading to early trial termination were encountered at a similar rate between elective and emergency studies. Similarly, there was no clear difference between citation numbers in emergency and elective trials, which was used as a marker for research impact. Variation between specialties revealed a comparable pattern to the overall picture.

Registration and publication of all surgical trials has previously been studied.(8) Emergency and elective trials were not studied separately, but found that 48% of all surgical trials remained published at 38 months. The current paper allowed a further 25 months minimum for our publication search and the overall publication proportion was similar (51.5%). This suggests a relative plateau in the rate of publication of the trials beyond 38 months. Interpretation of figure 2 however sees a plateau at around 55 months. Whilst more publications could be expected after this time, extending the search interval may limit applicability to current practice.

The discontinuation of RCTs in critical care has also been studied, which included seven surgical trials.(15) They found the percentage of 'acute care' trials (critical care or care within 24 hours of presentation) was 7%, and a similar publication rate between 'acute' and 'non-acute' trials. However, slow recruitment caused twice as many acute trials to be discontinued than non-acute trials. The methodology was based on comparing published protocols and publications, as opposed to trial registration and publications.

This study has several limitations. Emergency was a binary definition reflecting the wording used within the trials registries. There are inherent difficulties when trying to categorise patients this way, as some operations are 'urgent' but not 'emergency' (e.g. bone fractures). It is likely that within the trials searched, the definition of emergency varied. It is possible that this paper gives an overestimation of true emergency surgical trials, where 'urgent' trials have been upgraded to 'emergency' in attempting to define them.

Although this paper demonstrates a lack of registration of emergency surgical trials compared to surgical workload, further work is required to elicit the reasons. Because emergency trials are traditionally perceived as more difficult to achieve, both clinicians and sponsors may be more hesitant to invest their resources. This study shows that emergency surgical trials are equally as likely to be published as elective surgical trials and are a similar investment risk to elective randomised controlled trials. Indeed, many issues around consent and randomisation in emergency situations are being overcome.(16–20) With this

For peer review only - http://bmjopen.bmj.com/site/about/guidelines.xhtml

knowledge, clinicians, researchers and sponsors may feel reassured and inclined to be involved with emergency surgical trials.

Emergency surgery is under-represented in the literature, despite the high volume of workload it creates and poor outcomes. This study shows that registered emergency and elective surgical trials are equally as likely to be published with comparable recruitment issues. A perceived lack of successful completion and reporting of emergency surgical trials should no longer prevent investment into studies that may positively impact emergency surgery.

For peer review only

Figure Legend

Figure 1. PRISMA style flow diagram of search results, exclusions and group numbers.

Figure 2. Kaplan-Meier curves showing publication over time of emergency (blue) and elective (green) surgical trials. Only trials published on or after the July 2012 are shown.

For peer review only

References

1. Gale SC, Shafi S, Dombrovskiy VY, Arumugam D, Crystal JS. The public health burden of emergency general surgery in the United States. J Trauma Acute Care Surg [Internet]. 2014 Aug [cited 2017 Aug 3];77(2):202–8. Available from: http://www.ncbi.nlm.nih.gov/pubmed/25058242

2. RCSEng. Emergency Surgery - Standards for unscheduled surgical care [Internet]. 2011 [cited 2017 Oct 5]. Available from: https://www.google.co.uk/url?sa=t&rct=j&q=&esrc=s&source=web&cd=3&cad=rja&uact=8&ved=0ahUKEwiltJHc-JfXAhXBHxoKHcrzC7EQFggyMAI&url=https%3A%2F%2Fwww.rcseng.ac.uk%2F-%2Fmedia%2Ffiles%2Frcs%2Fabout-rcs%2Fregional%2Frcs_emergency_surgery_2011_web.pdf&usg=AOvV

3. McCord C, Ozgediz D, Beard JH, Debas HT. General Surgical Emergencies [Internet]. Essential Surgery: Disease Control Priorities, Third Edition (Volume 1). The International Bank for Reconstruction and Development / The World Bank; 2015 [cited 2017 Jul 3]. Available from: http://www.ncbi.nlm.nih.gov/pubmed/26741004

4. Mullen MG, Michaels AD, Mehaffey JH, Guidry CA, Turrentine FE, Hedrick TL, et al. Risk Associated With Complications and Mortality After Urgent Surgery vs Elective and Emergency Surgery. JAMA Surg [Internet]. 2017 May 10 [cited 2017 Jul 3]; Available from: http://www.ncbi.nlm.nih.gov/pubmed/28492821

5. Visser A, Geboers B, Gouma DJ, Goslings JC, Ubbink DT. Predictors of surgical complications: A?systematic review. Surgery [Internet]. 2015 Jul [cited 2017 Jul 5];158(1):58–65. Available from: http://linkinghub.elsevier.com/retrieve/pii/S0039606015000288

6. Saunders DI, Murray D, Pichel AC, Varley S, Peden CJ, UK Emergency Laparotomy Network. Variations in mortality after emergency laparotomy: the first report of the UK Emergency Laparotomy Network. Br J Anaesth [Internet]. 2012 Sep 1 [cited 2017 Nov 13];109(3):368–75. Available from: http://www.ncbi.nlm.nih.gov/pubmed/22728205

7. Becher RD, Hoth JJ, Miller PR, Mowery NT, Chang MC, Meredith JW. A critical assessment of outcomes in emergency versus nonemergency general surgery using the American College of Surgeons National Surgical Quality Improvement Program database. Am Surg [Internet]. 2011 Jul [cited 2017 Oct 5];77(7):951–9. Available from: http://www.ncbi.nlm.nih.gov/pubmed/21944366

8. Chapman SJ, Shelton B, Mahmood H, Fitzgerald JE, Harrison EM, Bhangu A. Discontinuation and non-publication of surgical randomised controlled trials: observational study. BMJ [Internet]. 2014 Dec 9 [cited 2016 Oct 31];349(dec09_1):g6870. Available from: http://www.bmj.com/content/349/bmj.g6870

For peer review only

9. Cook JA. The challenges faced in the design, conduct and analysis of surgical randomised controlled trials. Trials [Internet]. 2009 Dec 6 [cited 2017 Oct 19];10(1):9. Available from: http://www.ncbi.nlm.nih.gov/pubmed/19200379

10. BioMed Central Ltd. ISRCTN Registry [Internet]. [cited 2016 Oct 31]. Available from: http://www.isrctn.com/

11. U.S. National Library of Medicine. ClinicalTrials.gov [Internet]. [cited 2016 Oct 31]. Available from: https://clinicaltrials.gov/

12. Walker KF, Stevenson G, Thornton JG. Discrepancies between registration and publication of randomised controlled trials: an observational study. JRSM open [Internet]. SAGE Publications; 2014 May 1 [cited 2016 Oct 31];5(5):2042533313517688. Available from: http://shr.sagepub.com/content/5/5/2042533313517688.full

13. R Development Core Team (2008). A language and environment for statistical computing. R Foundation for Statistical Computing, Vienna, Austria [Internet]. p. http://www.r-project.org [accessed 13th Nov 2017]. Available from: http://www.r-project.org

14. Newcombe RG. TWO-SIDED CONFIDENCE INTERVALS FOR THE SINGLE PROPORTION: COMPARISON OF SEVEN METHODS. Stat Med Stat Med [Internet]. 1998 [cited 2018 Apr 23];17:857–72. Available from: http://citeseerx.ist.psu.edu/viewdoc/download?doi=10.1.1.408.7107&rep=rep1&type=pdf

15. Schandelmaier S, von Elm E, You JJ, Blümle A, Tomonaga Y, Lamontagne F, et al. Premature Discontinuation of Randomized Trials in Critical and Emergency Care. Crit Care Med [Internet]. 2016 Jan [cited 2017 Nov 13];44(1):130–7. Available from: http://www.ncbi.nlm.nih.gov/pubmed/26468895

16. Edwards M, Grocott M, Pearse R. Study Documents - FLuid Optimisation in Emergency LAparotomy trial [Internet]. 2017 [cited 2017 Nov 13]. Available from: http://www.floela.org/documents

17. Jansen J, Campbell M. UK-REBOA [Internet]. 2017 [cited 2017 Nov 13]. Available from: https://w3.abdn.ac.uk/hsru/REBOA/Public/Public/index.cshtml

18. Taylor J, Black S, J. Brett S, Kirby K, Nolan JP, Reeves BC, et al. Design and implementation of the AIRWAYS-2 trial: A multi-centre cluster randomised controlled trial of the clinical and cost effectiveness of the i-gel supraglottic airway device versus tracheal intubation in the initial airway management of out of hospital cardiac arrest. Resuscitation [Internet]. 2016 Dec [cited 2017 Nov 13];109:25–32. Available from: http://www.ncbi.nlm.nih.gov/pubmed/27697605

19. Roberts I, Shakur H, Coats T, Hunt B, Balogun E, Barnetson L, et al. The CRASH-2 trial: a randomised controlled trial and economic evaluation of the effects of tranexamic

For peer review only

acid on death, vascular occlusive events and transfusion requirement in bleeding trauma patients. Heal Technol Assess [Internet]. 2013 Mar [cited 2017 Aug 3];17(10):1–79. Available from: http://www.ncbi.nlm.nih.gov/pubmed/23477634

20. IMPROVE Trial Investigators I trial, Powell JT, Sweeting MJ, Thompson MM, Ashleigh R, Bell R, et al. Endovascular or open repair strategy for ruptured abdominal aortic aneurysm: 30 day outcomes from IMPROVE randomised trial. BMJ [Internet]. British Medical Journal Publishing Group; 2014 Jan 13 [cited 2017 Nov 13];348:f7661. Available from: http://www.ncbi.nlm.nih.gov/pubmed/24418950

For peer review only

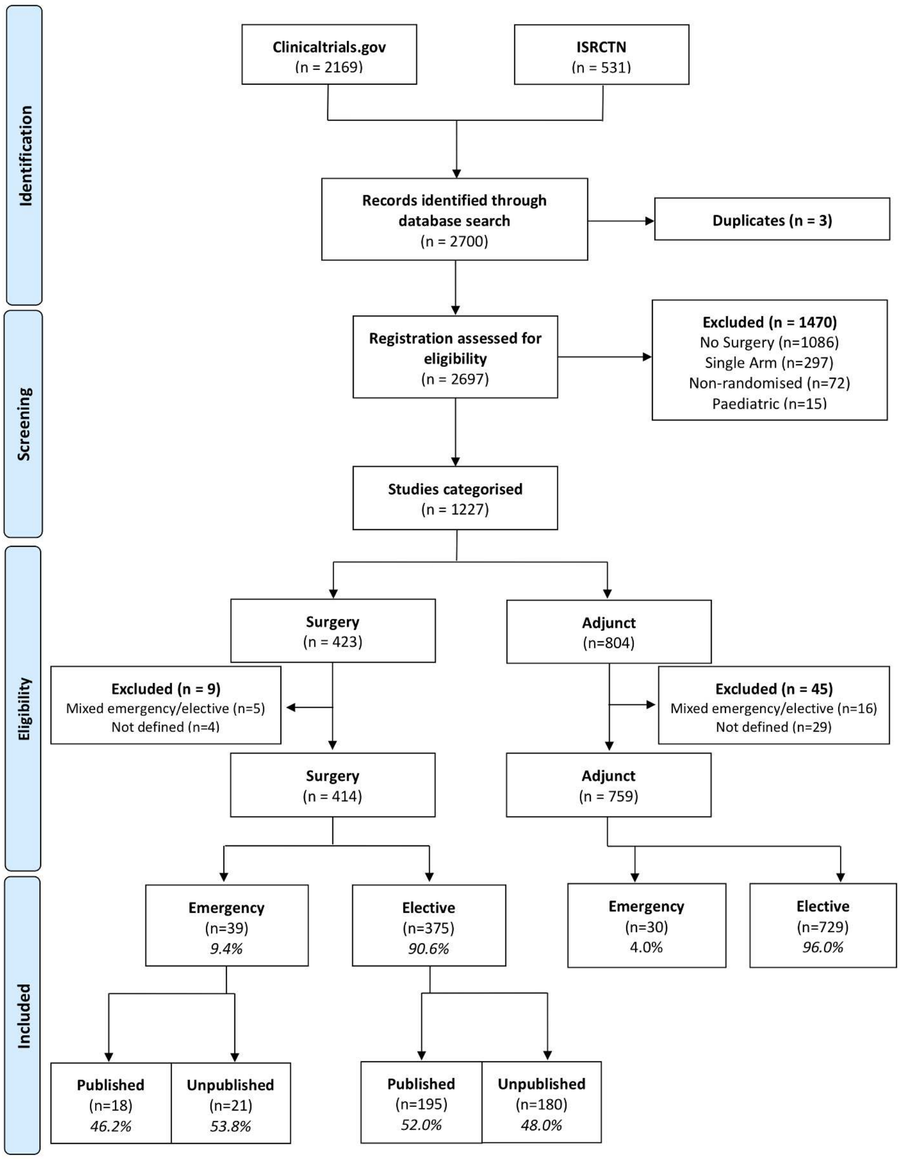

Figure 1. PRISMA style flow diagram of search results, exclusions and group numbers.

215x279mm (300 x 300 DPI)

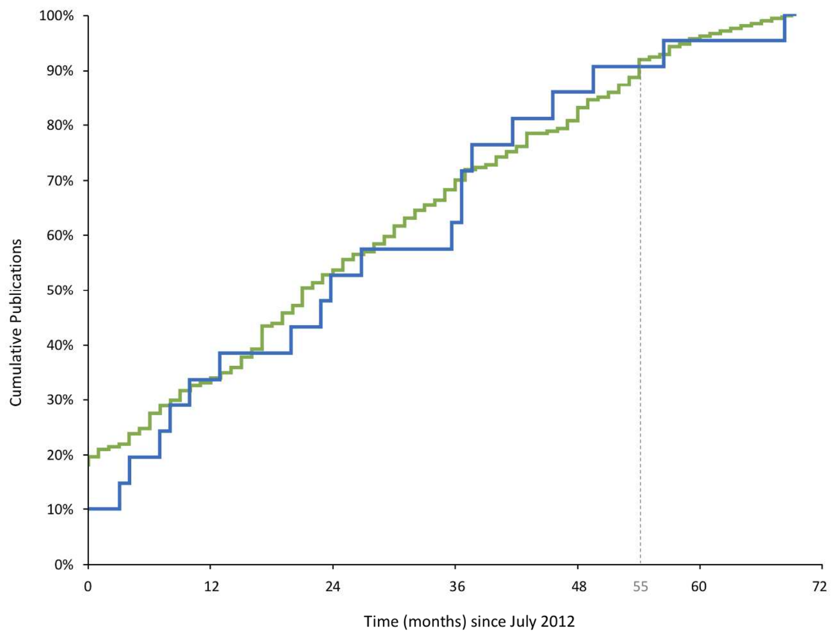

Figure 2. Kaplan-Meier curves showing publication over time of emergency (blue) and elective (green) surgical trials. Only trials published on or after the July 2012 are shown.

204x168mm (300 x 300 DPI)

Sponsorship	Emergency surgery; Number (% of total)	Elective surgery; Number (% of total)	All Number (% of total)
**Industry**	5 (12.8)	112 (29.9)	117 (28.6)
**Hospital**	18 (46.2)	120 (32)	138 (33.3)
**University**	10 (25.6)	92(24.5)	102 (24.6)
**Government**	5 (12.8)	21 (5.6)	26 (6.3)
**Research institute**	1 (2.6)	30 (8.0)	31 (7.5)
**TOTAL**	39 (100)	375 (100)	414 (100)

Supplementary Table 1 Comparison of the origin of sponsorship between emergency and elective surgical trials. Decimals do not add up exactly to total due to rounding.

For peer review only
